# Self-similar transport, spin polarization and thermoelectricity in complex silicene structures

**DOI:** 10.1038/s41598-020-71697-1

**Published:** 2020-09-07

**Authors:** R. Rodríguez-González, L. M. Gaggero-Sager, I. Rodríguez-Vargas

**Affiliations:** 1 CIICAp, IICBA, Universidad Autónoma del Estado de Morelos, Av. Universidad 1001, Col. Chamilpa, 62209 Cuernavaca, Morelos Mexico; 2 Unidad Académica de Ciencia y Tecnología de la Luz y la Materia, Universidad Autónoma de Zacatecas, Carretera Zacatecas-Guadalajara Km. 6, Ejido La Escondida, 98160 Zacatecas, Zac. Mexico

**Keywords:** Two-dimensional materials, Condensed-matter physics, Electronic properties and materials

## Abstract

2D materials open the possibility to study Dirac electrons in complex self-similar geometries. The two-dimensional nature of materials like graphene, silicene, phosphorene and transition-metal dichalcogenides allow the nanostructuration of complex geometries through metallic electrodes, interacting substrates, strain, etc. So far, the only 2D material that presents physical properties that directly reflect the characteristics of the complex geometries is monolayer graphene. In the present work, we show that silicene nanostructured in complex fashion also displays self-similar characteristics in physical properties. In particular, we find self-similar patterns in the conductance, spin polarization and thermoelectricity of Cantor-like silicene structures. These complex structures are generated by modulating electrostatically the silicene local bandgap in Cantor-like fashion along the structure. The charge carriers are described quantum relativistically by means of a Dirac-like Hamiltonian. The transfer matrix method, the Landauer–Büttiker formalism and the Cutler–Mott formula are used to obtain the transmission, transport and thermoelectric properties. We numerically derive scaling rules that connect appropriately the self-similar conductance, spin polarization and Seebeck coefficient patterns. The scaling rules are related to the structural parameters that define the Cantor-like structure such as the generation and length of the system as well as the height of the potential barriers. As far as we know this is the first time that a 2D material beyond monolayer graphene shows self-similar quantum transport as well as that transport related properties like spin polarization and thermoelectricity manifest self-similarity.

## Introduction

The discovery of 2D materials has attracted a great interest in the scientific community, giving rise to a new era in materials science^1^. Despite the widespread family of 2D materials discovered so far, monolayer graphene is undoubtedly one of the most studied materials due to the multitude of exotic properties that sustains and the great technological potential that it possesses. With regard to exotic properties, these have been theoretically studied and experimentally observed. Among the most remarkable properties we can find the so-called Klein tunneling^2,3^, atomic collapse^4^ and the Hofstadter’s butterfly^5,6^.

Recently, it has been reported that charge carriers manifest quantum fractal nature once they are confined in structures with fractal geometries^7–12^. Fractals are objects with special properties such as fractal dimension and self-similarity^13^. Self-similar geometries self-repeat its whole shape at all scales. Typical self-similar geometries are based on the Sierpinski carpet, Sierpinski triangle, Vicsek fractal and Cantor set^14^. Under this context, theoretical studies on quantum transport have been carried out in complex (self-similar) graphene structures^7,8^. In particular, the quantum conductance has been studied in a complex geometry based on the Sierpinski carpet^7^. The box-counting method has been implemented to calculate the fractal dimension of the conductance fluctuations. The resulting dimension inherits the fractal dimension of the Sierpinski carpet in question, being this behavior an intrinsic property of the geometrical construction of each Sierpinski carpet iteration. In other words, the fractal dimension of the conductance fluctuations can be tuned by changing the geometry of the self-similar structure. From the experimental standpoint, self-similar geometries have also been explored. For instance, Kempkes et al.^15^ have applied atomic manipulation through scanning tunnelling microscopy to create an artificial version of different generations of the Sierpinski triangle. The fractal dimension of the electronic wavefunctions has been measured, finding that it corresponds to the dimension of the Sierpinski triangle, showing that electrons live in a non-integer dimensional space. Furthermore, it has been reported that the transport properties in graphene manifest self-similar characteristics when the graphene sheet is placed on substrates that are nanostructured according to a variation of the common Cantor set^8^. In this case, the factor three typical of the Cantor set is crucial for scaling both the height and length of the barriers. In particular, self-similar patterns in the conductance that are well described by scaling rules are reported. Here, it is important to mention that substrates with different degrees of interaction with the graphene sheet (different barrier heights) are needed in order to obtain the self-similar graphene structures. It is also reported that simpler complex graphene structures present self-similar physical properties^16–19^. In particular, self-similar graphene structures, in which only the length of the barriers is scaled according to the Cantor set rules, display self-similar characteristics in the transmission probability or transmittance. These self-similar structures can be generated by nanostructured substrates^16–18^ and inhomogeneous magnetic fields^19^. As we have documented graphene is the only material that manifests a fractal complex behavior in the electron transport. So, the so-called self-similar transport can be considered as an additional exotic or peculiar phenomenon in graphene as the ones mentioned above.

Regarding silicene^20–23^, a graphene analogue with silicon atoms, it is reported that owns plethora of exotic phenomena. The low-buckled crystal structure and the large spin-orbit coupling (SOC)^24,25^ of the material give rise to a valley-spin dependent band structure with a local bandgap modulable with an external electric field^26,27^. This special band structure allows multiple topological phases in silicene^27–33^. Electric, magnetic and optical fields as well as doping can induce phases like valley-polarized metal, quantum anomalous Hall effect, quantum spin Hall effect and chiral topological superconductivity. Furthermore, silicene exhibits a highly fragmented energy spectrum with fractal characteristics (Hofstadter butterfly) under strong perpendicular magnetic field and a periodic potential^34^. The fractal patterns depend on the SOC as well as the external perpendicular electric field. Fractal butterflies are also presented in silicene with magnetic order^35^. The topological phases are characterized by high spin-Chern numbers giving rise to the so-called high spin-Chern insulators. So, as we have presented the fractal energy spectra of silicene are well documented. However, as far as we know, there are no reports about the physical properties of silicene under complex geometries. In particular, studies that assess the impact of complex nanostructuration on the transport and transport-related properties. Taking into account the relevance of silicene from both the theoretical and experimental standpoint, we consider that further studies about the physical properties of complex silicene structures are required.

In this work, we try to fill this gap by studying the transport, spin-polarization and thermoelectric properties of complex silicene structures. We consider complex structures, self-similar potential profiles, generated through metallic electrodes arranged in Cantor-like fashion over silicene. We adopt a quantum relativistic description for the silicene charge carriers. The transfer matrix method, the Landauer–Büttiker formalism and the Cutler–Mott formula are used to obtain the transmission, transport and thermoelectric properties, respectively. Our results indicate that transport and transport-related quantities like the conductance, spin polarization and Seebeck coefficient show self-similar characteristics. The main parameters of the complex structure such as the generation number, the height of the barriers and the length of the system are involved in the self-similar patterns. In addition, we quantify numerically the self-similar patterns with well-defined scaling rules. To our knowledge, this is the first time that self-similar transport is reported beyond graphene. More importantly, that relevant quantities like spin polarization and the Seebeck coefficient manifest self-similar characteristics.

**Figure 1:**
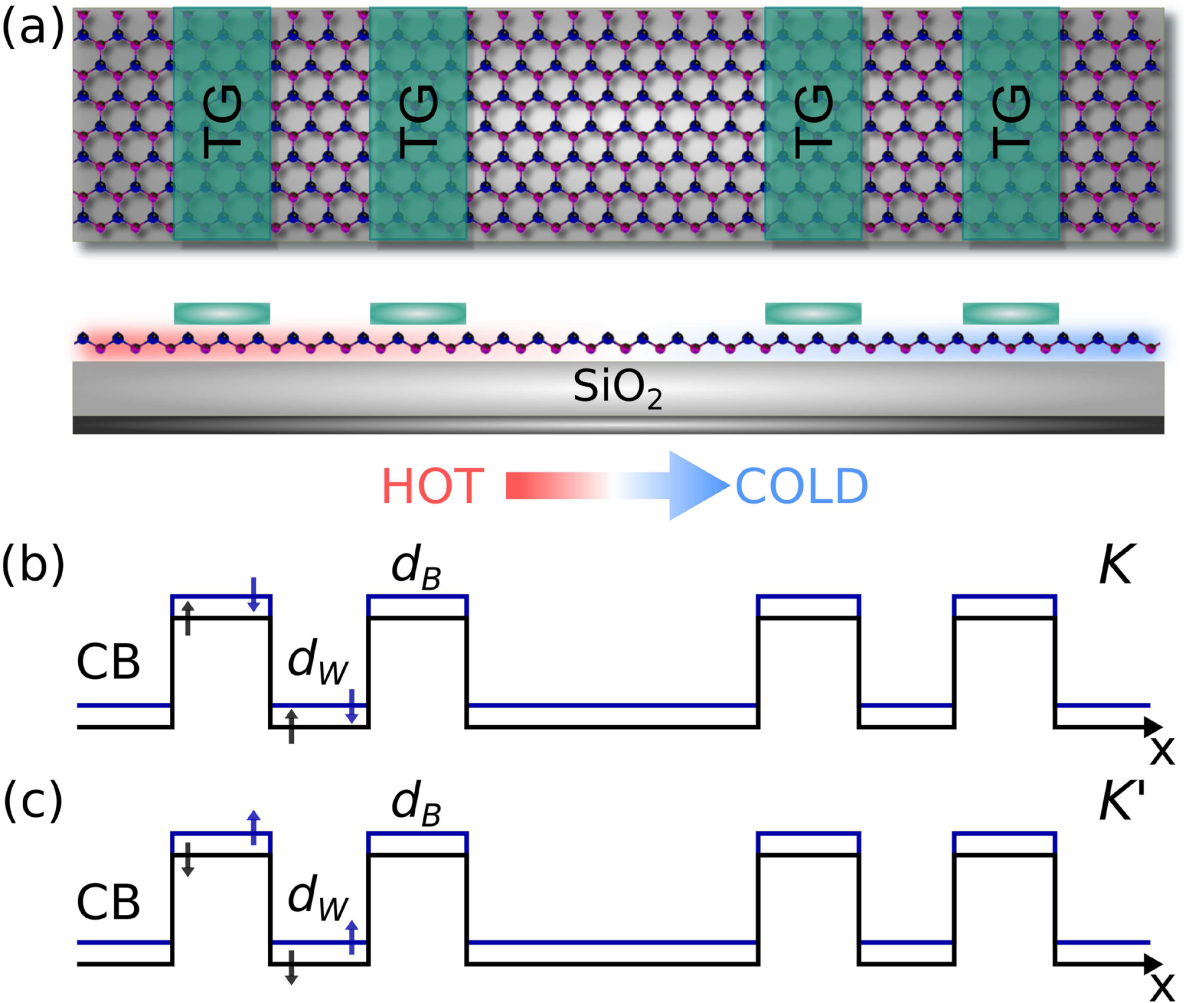
(**a**) Schematic representation for Cantor-like silicene structures, a top and side view are depicted. The silicene sheet is placed above of a dielectric substrate like ${\hbox {SiO}}_2$. To achieve a complex geometry the metallic electrodes or also named as top gates (TGs) are arranged in a Cantor-like fashion along the structure. In the TGs an external electrostatic potential is applied to modulate the local bandgap. In the side view, the possible thermoelectric device is depicted. A temperature gradient is established from left to right, with the arrow indicating the heat flux. (**b**) Band-edge profiles for the conduction band (CB) in the *K* valley. The black and blue lines stand for spin up and down orientation, respectively. $d_W$ and $d_B$ represent the width of well and barrier regions. (**c**) The same as (**b**) but now the $K'$ valley is illustrated, where the spin orientation is reversed. This scheme corresponds to the third generation *N*3 according to the Cantor sequence.

## Methodology

To illustrate in detail the system we are interested in, we will first address its fundamental characteristics as well as the theoretical approach used to obtain the transmission, transport and thermoelectric properties. The silicene Cantor structures are mainly conformed by a silicene sheet along with metallic electrodes. The silicene is placed on a non-interacting substrate like ${\hbox {SiO}}_{2}$. In order to have a complex structure the metallic electrodes are incorporated in a fractal geometric distribution like the triadic Cantor set as depicted in Fig. 1a. A perpendicular electrostatic field $E_{z}$ is applied through metallic electrodes giving rise to a complex barrier arrangement. Furthermore, hot and cold contacts have been added at the ends of the system to generate a temperature gradient with the aim to study thermoelectricity, see the bottom of Fig. 1a. A quantum relativistic description has been adopted for the charge carriers in silicene. Therefore, we need to solve a Dirac-like eigenvalue equation for the following low-energy effective Hamiltonian^25,36,37^,

1 $$\begin{aligned} H=\hbar v_{F}(k_{x}\tau _{x}-\eta k_{y}\tau _{y}) - (\eta \sigma \Gamma _{SO}-\Delta _{z})\tau _{z}, \end{aligned}$$

where $v_{F}$ is the Fermi velocity, $k_{x}$ and $k_{y}$ are the components of the wave vector in the *xy* plane, $\tau _{x}$, $\tau _{y}$ and $\tau _{z}$ are the Pauli matrices in the sublattice pseudospin space, $\eta =\pm 1$ is the valley index and represents the *K* and $K'$ valley, $\sigma =\pm 1$ indicates the electron spin components, $\Gamma _{SO}=3.9$ meV is the spin-orbit interaction in silicene, $\Delta _{z}$ corresponds to the on-site potential difference between the two sublattices A and B of the silicene hexagonal structure. Here, $E_{z}$ can modulate $\Delta _{z}$ and consequently the silicene local bandgap.

**Figure 2:**
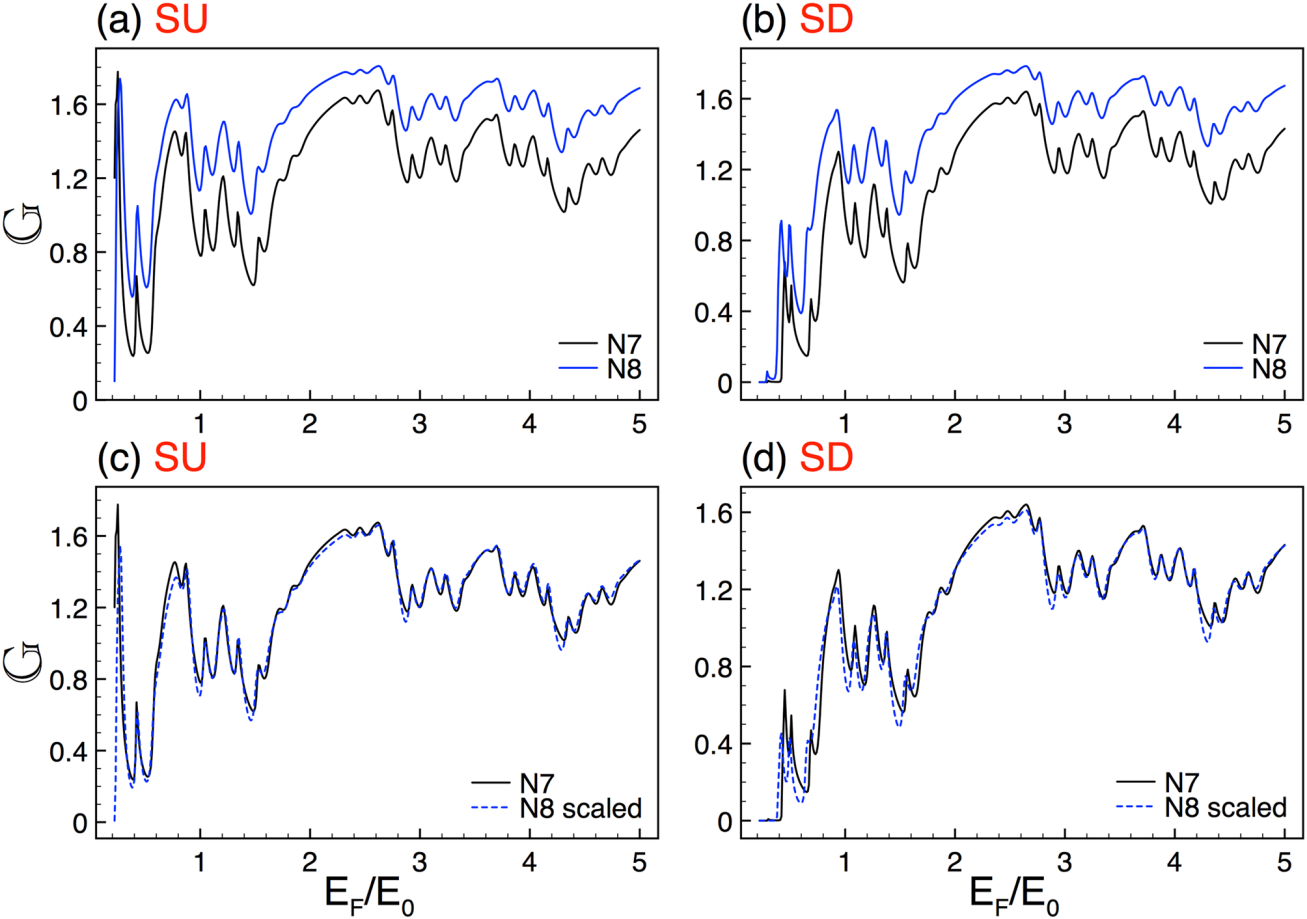
Conductance scaling between generations for SU and SD. (**a**) and (**b**) represent the conductance curves for the seventh *N*7 (solid-black lines) and eighth *N*8 (solid-blue lines) generations. (**c**) and (**d**) are the same as in (**a**) and (**b**), but here *N*8 (dashed-blue lines) is scaled according to Eq. (13), with $\alpha _{+1}=1.83$ and $\alpha _{-1}=1.89$. The other structural parameters are $\Delta =2$ and $w=30$.

Take into account the $\eta$ valley and $\sigma$ spin indexes as well as the specific *j*th region, the wave functions for this Hamiltonian can be written as,

2 $$\begin{aligned} \psi _{j}(x,y) = \left[ A^{\eta ,\sigma }_{j} \left( \begin{array}{c} 1 \\ v^{\eta ,\sigma }_{j,+} \end{array}\right) e^{ik_{x,j}x} + B^{\eta ,\sigma }_{j} \left( \begin{array}{c} 1 \\ v^{\eta ,\sigma }_{j,-} \end{array}\right) e^{-ik_{x,j}x} \right] e^{ik_{y,j}y}, \end{aligned}$$

where the subscript *j* stands for the wave functions in each region, either potential barrier or well. Here $A^{\eta ,\sigma }_{j}$, $v^{\eta ,\sigma }_{j,+}$ and $B^{\eta ,\sigma }_{j}$, $v^{\eta ,\sigma }_{j,-}$ belong to the incident and reflected waves, respectively. Moreover, the bispinor coefficients come as,

3 $$\begin{aligned} v^{\eta ,\sigma }_{j,\pm } = \frac{\hbar v_{F}(\pm k_{x,j}-i\eta k_{y,j})}{E-\eta \sigma \Gamma _{SO}+\Delta _{z,j}}, \end{aligned}$$

with the corresponding wave vectors given as,

4 $$\begin{aligned} k_{x,j}=\frac{1}{\hbar v_F} \sqrt{E^2-(\eta \sigma \Gamma _{SO} - \Delta _{z,j})^2 - (\hbar v_F)^2 k_{y,j}^2}. \end{aligned}$$

**Figure 3:**
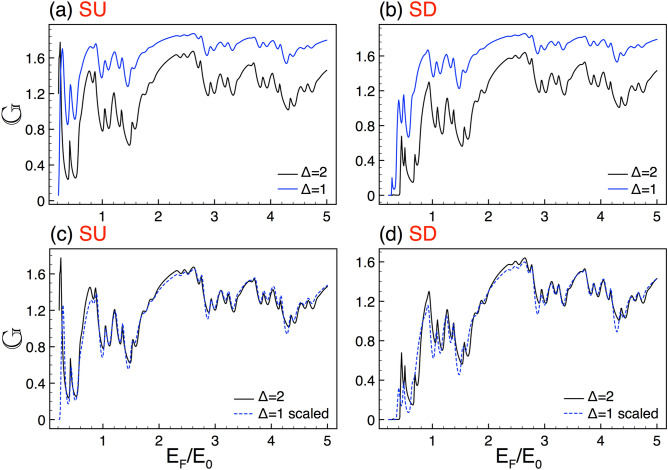
Conductance scaling between barrier heights for SU and SD. (**a**) and (**b**) illustrate the conductance curves for $\Delta =2$ (solid-black lines) and $\Delta =1$ (solid-blue lines). (**c**) and (**d**) are the same as in (**a**) and (**b**), but now the spectrum of $\Delta =1$ (dashed-blue lines) is transformed with the help of Eq. (14), where $\beta _{+1}=2.87$ and $\beta _{-1}=3.02$. The other structural parameters are *N*7 and $w=30$.

As there is conservation of momentum in the transverse direction, thus $k_{y,j}=k_{y}$ for all regions, with $k_y=k\sin \theta$ and $k=(\hbar v_F)^{-1}\sqrt{E^2 - (\eta \sigma \Gamma _{SO})^2}$. From now on, we have used for the barrier region the notation $\Delta _{z,j}=\Delta$, while for the well regions $\Delta _{z,j}=0$. In addition, we will introduce parameters as the characteristic length $a_{0}=20$ nm and its corresponding energy $E_{0}=\hbar v_F/a_{0}=18.1$ meV in order to express the length and energy quantities in dimensionless units.

**Figure 4:**
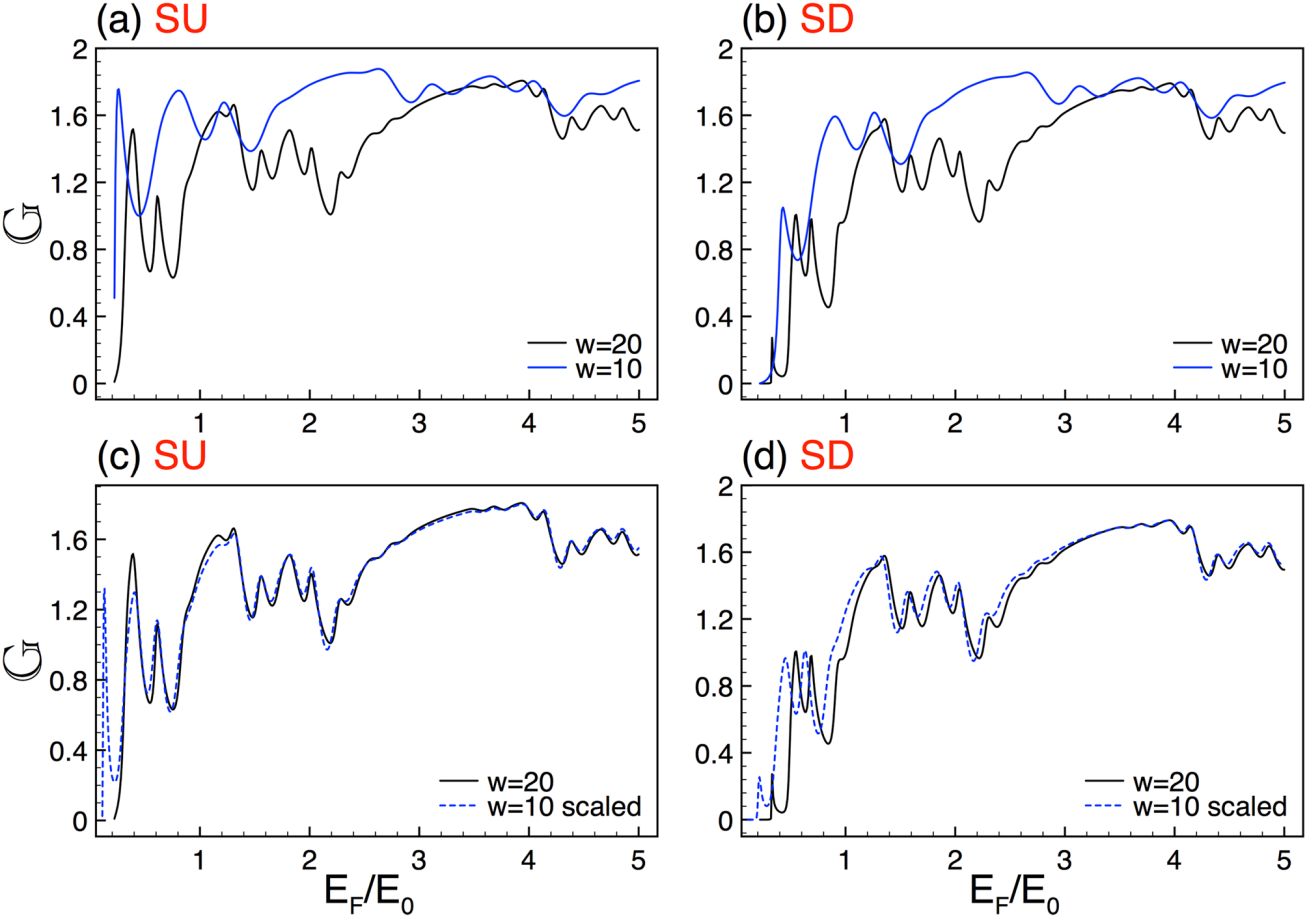
Conductance scaling between lengths for SU and SD. (**a**) and (**b**) stand for the conductance curves for $w=20$ (solid-black lines) and $w=10$ (solid-blue lines). (**c**) and (**d**) are the same as in (**a**) and (**b**), but the curve of $w=10$ (dashed-blue lines) is scaled applying Eq. (15), with $\gamma _{+1}=\gamma _{-1}=3.2$. The other structural parameters are *N*7 and $\Delta =2$.

Once that we have defined the eigenfunctions and eigenvalues of the above Hamiltonian and taking into account the wave function continuity along the *x* direction in each interface, $\psi _{j}(x_j,y)=\psi _{j+1}(x_{j},y)$, of the complex structure, we can compute the transmission probability or transmittance of each valley-spin channel through the so-called transfer matrix^38,39^,

5 $$\begin{aligned} T^{\eta ,\sigma }=\left| \frac{A^{\eta ,\sigma }_{N+1}}{A^{\eta ,\sigma }_0} \right| ^{2} = \frac{1}{| M^{\eta ,\sigma }_{11} |^2}. \end{aligned}$$

**Figure 5:**
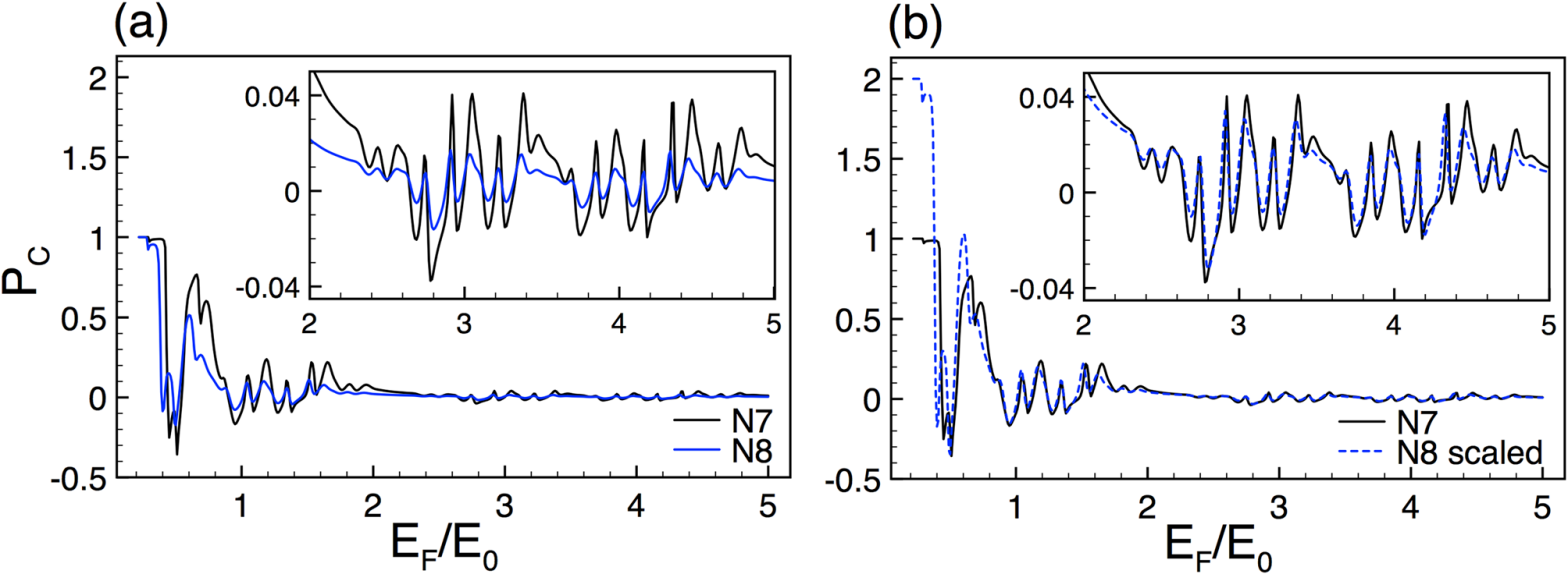
Conductance spin polarization scaling between generations. (**a**) Spin polarization curves for the seventh *N*7 (solid-black lines) and eighth *N*8 (solid-blue lines) generations. (**b**) The same as in (**a**), but here Eq. (16) is used to obtain the scaled generation *N*8 (dashed-blue lines). The subview in (**b**) illustrates more clearly the scalability. The rest of the structural parameters are the same as in Fig. 2.

Here, we take advantage of the relationship between the coefficients of the initial medium $A^{\eta ,\sigma }_{0}$ and $B^{\eta ,\sigma }_{0}$ and those of the final one $A^{\eta ,\sigma }_{N+1}$ and $B^{\eta ,\sigma }_{N+1}=0$,

6 $$\begin{aligned} \left( \begin{array}{c} A^{\eta ,\sigma }_0 \\ B^{\eta ,\sigma }_0 \end{array}\right) = \left( \begin{array}{c} M^{\eta ,\sigma }_{11} \quad M^{\eta ,\sigma }_{12}\\ M^{\eta ,\sigma }_{21} \quad M^{\eta ,\sigma }_{22} \end{array}\right) \left( \begin{array}{c} A^{\eta ,\sigma }_{N+1} \\ 0 \end{array}\right) , \end{aligned}$$

with the transfer matrix

7 $$\begin{aligned} M^{\sigma , \eta }=D_{0}^{-1}\left[ \prod _{j=1}^{N}D_{j}P_{j}D_{j}^{-1}\right] D_{0}. \end{aligned}$$

given in terms of the dynamic matrices

8 $$\begin{aligned} D_j= \left( \begin{array}{cc} 1 &{} 1 \\ v^{\eta ,\sigma }_{j,+} &{} v^{\eta ,\sigma }_{j,-} \end{array} \right) , \end{aligned}$$

and the propagation ones

9 $$\begin{aligned} P_j= \left( \begin{array}{cc} e^{-ik_{x,j}(x_{j}-x_{j-1})} &{} 0 \\ 0 &{} e^{ik_{x,j}(x_{j}-x_{j-1})} \end{array} \right) . \end{aligned}$$

**Figure 6:**
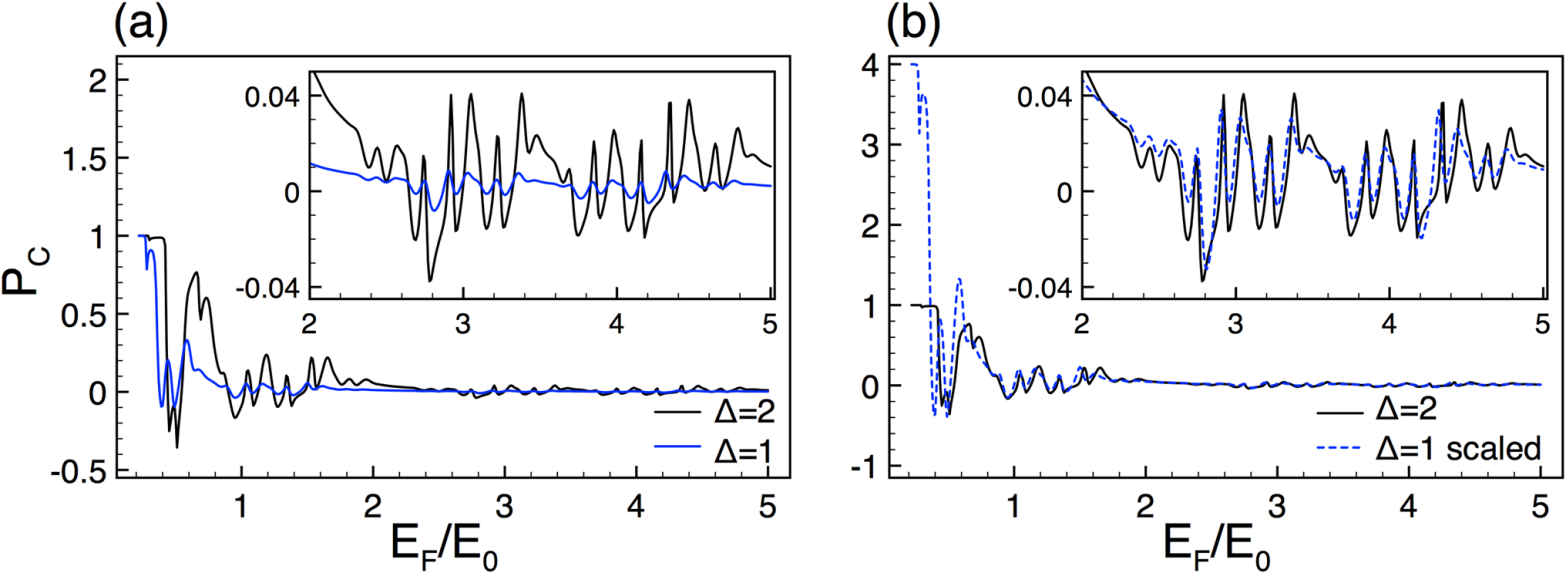
Conductance spin polarization scaling between barrier heights. (**a**) Spin polarization curves for $\Delta =2$ (solid-black lines) and $\Delta =1$ (solid-blue lines). (**b**) The same as in (**a**), but here the curve of $\Delta =1$ (dashed-blue lines) is transformed according to Eq. (17). For more details see the subviews. The rest of the structural parameters are the same as in Fig. 3.

The difference $x_{j}-x_{j-1}$ represents the width of the *j*th region. Moreover, in the present case $D_0$, which represents the dynamic matrix of the semi-infinite left and right regions, is equivalent to the matrices of the well regions.

**Figure 7:**
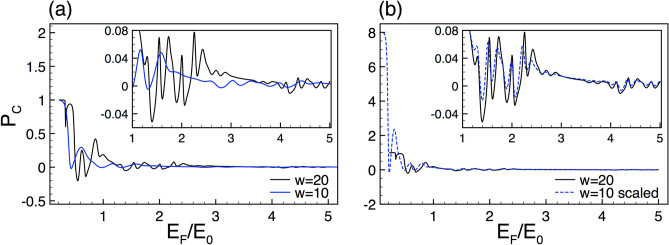
Conductance spin polarization scaling between lengths. (**a**) Spin polarization curves for $w=20$ (solid-black lines) and $w=10$ (solid-blue lines). (**b**) The same as in (**a**), but here the curve associated to $w=10$ (dashed-blue lines) is scaled according to Eq. (18). The subview in (**b**) depicts more details about the scaling. The rest of the structural parameters are the same as in Fig. 4.

Now, it is possible to deduce for a specific channel ($\eta$, $\sigma$) the ballistic conductance through the complex multi-barrier structures via the Landauer–Bütikker formula^40^, namely,

10 $$\begin{aligned} G^{\eta ,\sigma }(E_{F})=G_{0} \int ^{\pi /2}_{-\pi /2}T^{\eta ,\sigma }(E_{F},\theta )\cos \theta d\theta , \end{aligned}$$

with $E_{F}$ the Fermi energy, $G_0=e^{2}L_{y}k_{F}/\pi ^{2}\hbar$ the fundamental unit of conductance, $L_y$ the system transverse length (*y* direction), $k_{F}=\sqrt{E^{2}_{F}-\Gamma ^{2}_{SO}}$ the Fermi wave vector and $\theta$ the incident angle of charge carriers with respect to the propagation direction (*x* direction).

**Figure 8:**
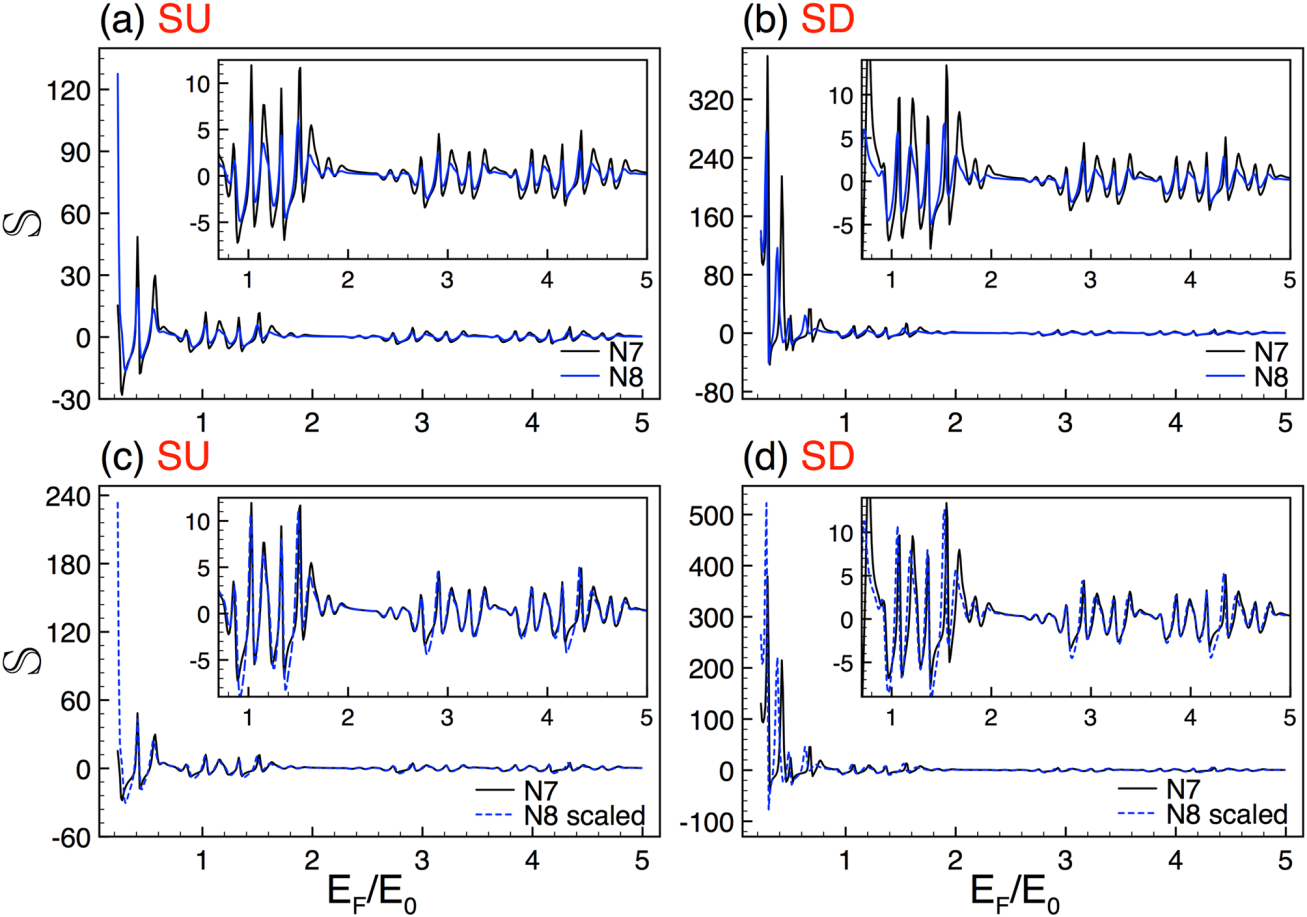
Scaling between generations for the Seebeck coefficient. The scaled curve is obtained using Eq. (21). The average temperature considered is 50 K. The system parameters are the same as in Fig. 2.

Likewise, we can compute the conductance spin polarization $P_C$ for the *K* or $K'$ valley,

11 $$\begin{aligned} P^{\eta }_C=\dfrac{G^{\eta ,+} - G^{\eta ,-}}{G^{\eta ,+} + G^{\eta ,-}}. \end{aligned}$$

**Figure 9:**
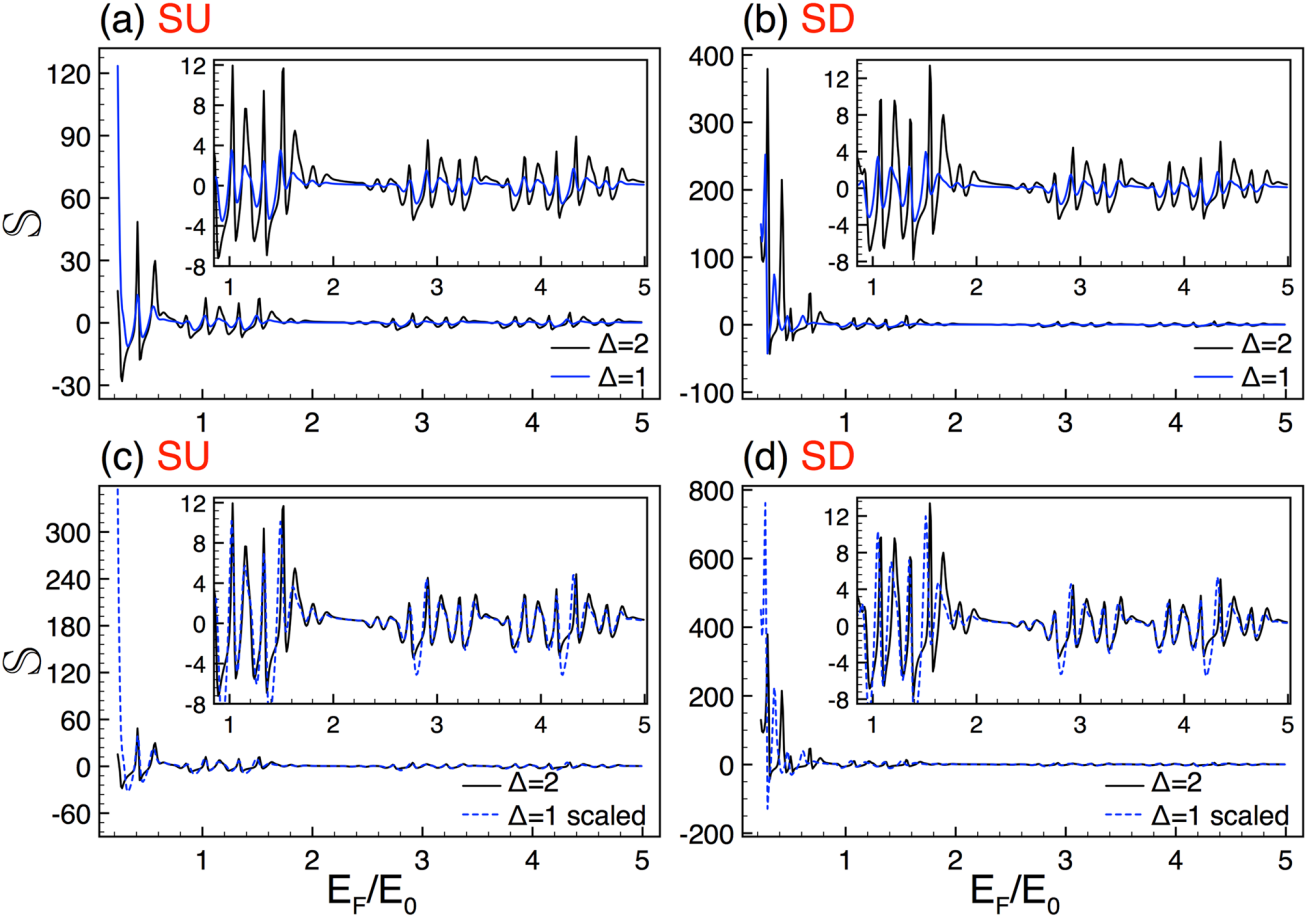
Scaling between barrier heights for the Seebeck coefficient. The scaled curve is obtained using Eq. (22). The average temperature considered is 50 K. The system parameters are the same as in Fig. 3.

Finally, as a temperature gradient along our system is generated it is possible to study the thermoelectric effects. To do so, we consider the Cluter–Mott relation^41^, which allows us to compute the low-temperature Seebeck coefficient as

12 $$\begin{aligned} \left. S^{\eta ,\sigma }(E)=\dfrac{\pi ^{2} k_{\beta }^{2} T}{3e}\dfrac{\partial \ln [ G^{\eta ,\sigma }(E)]}{\partial E} \right| _{E=E_F}, \end{aligned}$$

where $k_{\beta }$ is the Boltzmann constant, *T* is the average temperature between the hot and cold contacts and *e* is the bare electron charge.

**Figure 10:**
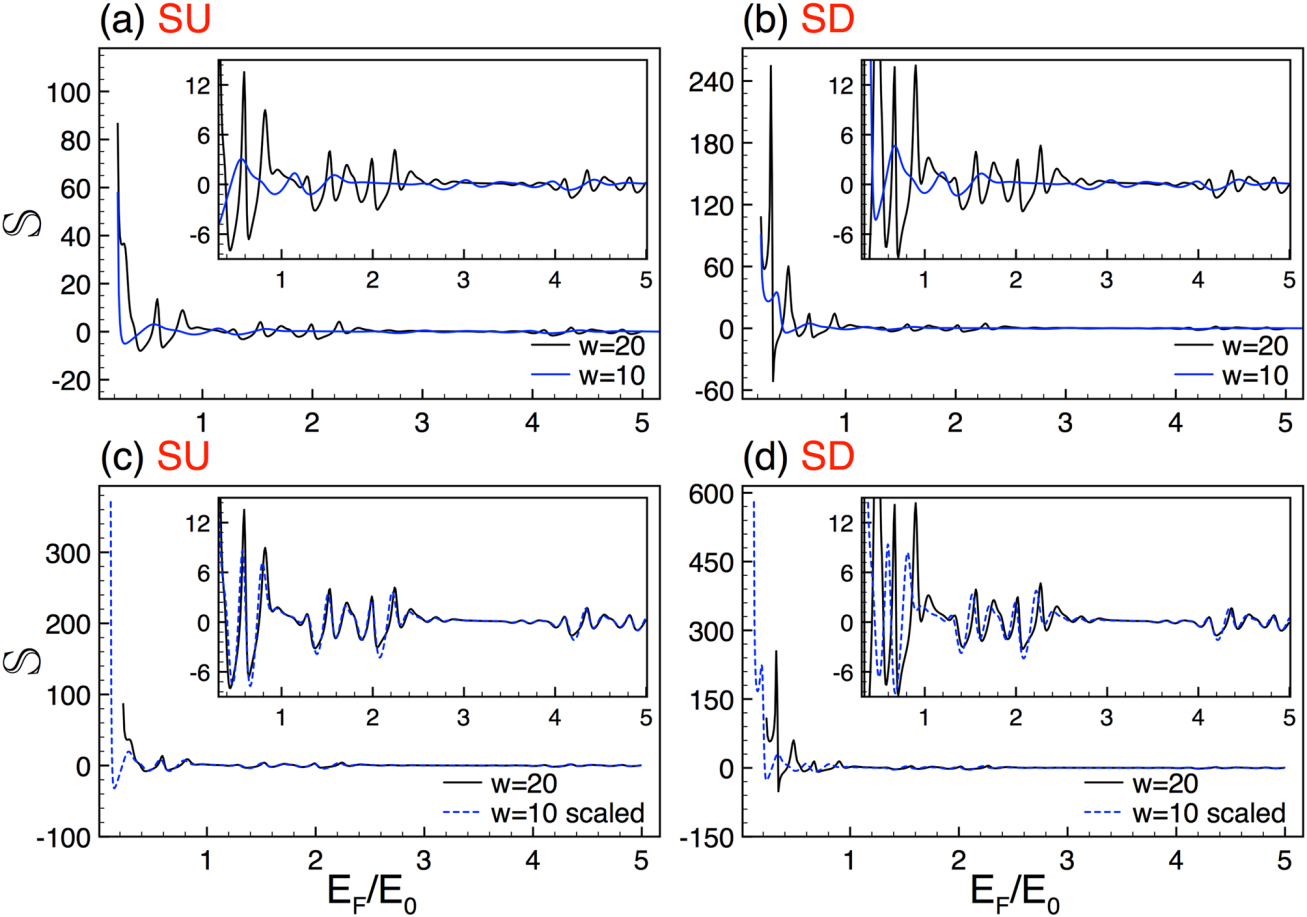
Scaling between lengths for the Seebeck coefficient. The scaled curve is obtained using Eq. (23). The average temperature considered is 50 K. The system parameters are the same as in Fig. 4.

## Results

In this section we present the main results about the transport properties, the spin polarization and the thermoelectric effects in complex silicene structures. In particular, we show that these properties display a self-similar behavior for both spin orientation: spin up (SU) and down (SD). Moreover, we will characterize mathematically the self-similar patterns by deriving scaling expressions numerically in the case of the conductance and spin polarization and analytically for the Seebeck coefficient. We will also show, as in the case of graphene complex structures^8^, that structural parameters such as the generation of the Cantor-like structure *N*, the height of the barriers $\Delta$ and the length of the system *w* are directly involved in the scaling expressions (self-similar patterns). It is important to remark that all our numerical results are computed for the *K* valley ($\eta =+1$), since the results for the $K'$ valley ($\eta =-1$) can be obtained straightforwardly by simply reversing the spin orientation, see Fig. 1b,c. So, from now on, we will suppress the valley index $\eta$ in the scaling expressions.

**Figure 11:**
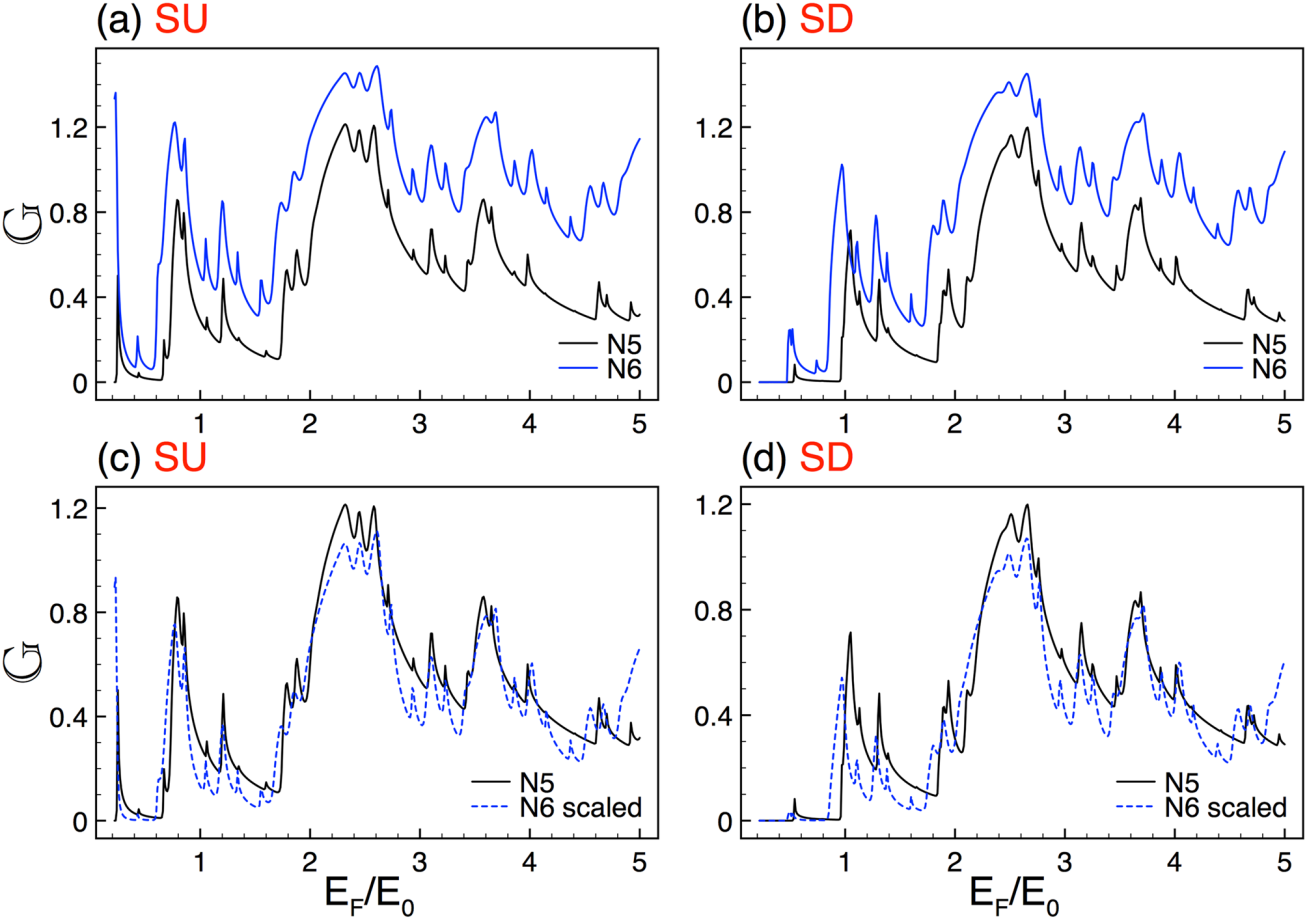
The same as Fig. 2, but here the generations are *N*5 and *N*6, reference and scaled curve, respectively. In this case the scale factors take values $\alpha _{+1}=1.98$ and $\alpha _{-1}=1.95$.

### Transport properties

In first place, we address the self-similar patterns that arise in the linear-regime conductance once silicene is nanostructured in Cantor-like fashion. As we can see in Fig. 2 the conductance curves of different generations of the Cantor-like structure have similar envelopes. The similarity is presented for both spin components, SU and SD, Fig. 2a,b, respectively. The main difference between the conductance curves is an offset in the vertical axis, which tells us that we can go from one curve to the other with an appropriate scale factor. Determining the scale factors of the self-similar conductance patterns can be tricky. However, we can obtain them by following the protocol reported for graphene complex structures^8^. In particular, we can use an auxiliary conductance-related quantity to unravel the precise scale factors. The guidelines of the protocol and specific results of the auxiliary quantity for complex silicene structures can be found in the Supplementary information. So, in the rest of this subsection we will focus on the conductance scaling rules without forgetting that there is a protocol to obtain them. In the case of the self-similar patterns between generations the scaling expression is given as

13 $$\begin{aligned} {\mathbb {G}}^{\sigma }_{N}(E_{F})\approx \frac{\left[ {\mathbb {G}}^{\sigma }_{N+1}(E_{F})\right] ^{\alpha _{\sigma }}}{(2)^{\alpha _{\sigma }-1}}, \end{aligned}$$

where the exponent $\alpha _{\sigma }$ depends on the structural parameters and the spin component and *N* represents the generation number. The conductance in this expression and the coming ones is given in terms of the fundamental conductance factor, ${\mathbb {G}}^{\sigma }(E_F)=G^{\sigma }(E_F)/G_{0}$. In the specific cases shown in Fig. 2 the exponents take values of $\alpha _{+1}=1.83$ and $\alpha _{-1}=1.89$. The results of the scaling can be appreciated in Fig. 2c,d. As we can see the scaled curves *N*8 (dashed-blue lines) match quite well with the reference ones *N*7 (solid-black lines). Here, we would like emphasize that the exponents of the spin components are not equivalent because the corresponding conductance curves are dissimilar. This is related to the spin-dependent silicene band structure, which results in fundamental differences between the complex barrier structures for SU and SD.

**Figure 12:**
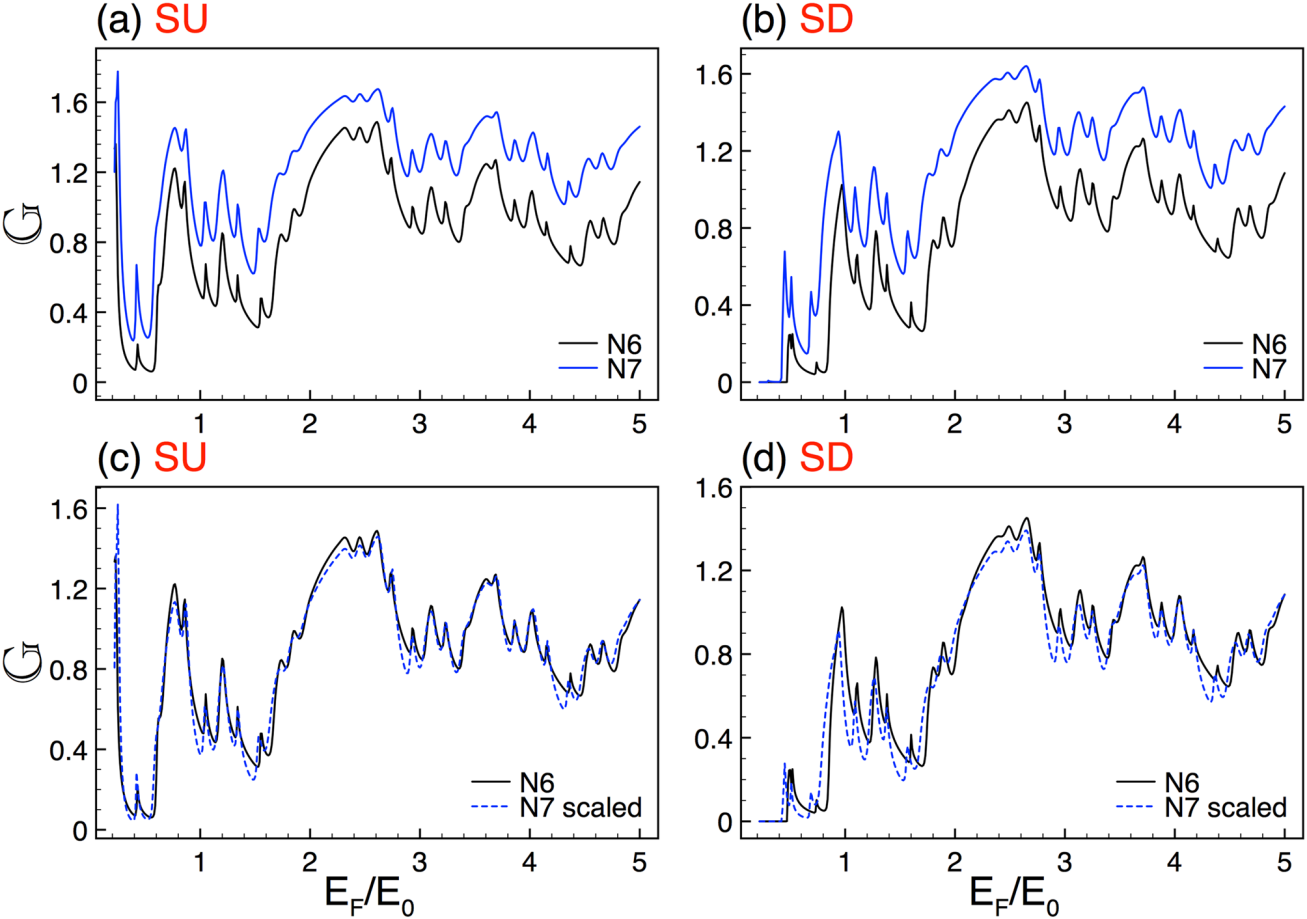
The same as Fig. 2, but here the generations are *N*6 and *N*7, reference and scaled curve, respectively. In this case the scale factors take values $\alpha _{+1}=1.78$ and $\alpha _{-1}=1.83$.

**Figure 13:**
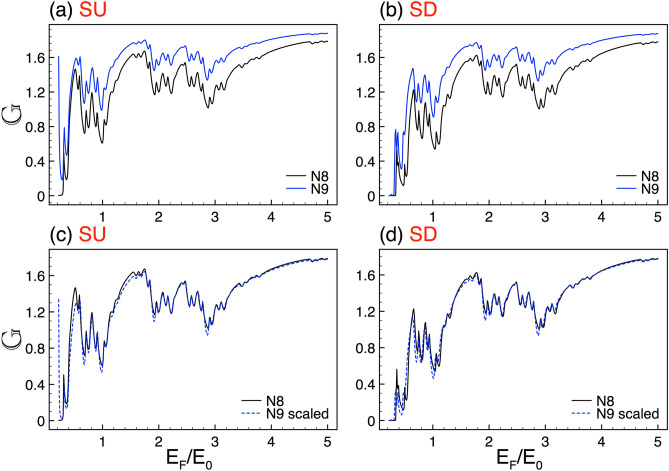
The same as Fig. 2, but here the generations are *N*8 and *N*9, reference and scaled curve, respectively. In this case the scale factors take values $\alpha _{+1}=1.87$ and $\alpha _{-1}=1.86$. Here the structural parameters are $\Delta =2$ and $w=45$.

Now, it is turn to explore the scaling associated to the height of the barriers. When we consider complex silicene structures with different heights in the barriers the conductance curves look quite similar. See for instance the results (Fig. 3) for complex structures with barriers of $\Delta =2$ and $\Delta =1$. In these cases the generation and the length of the system are *N*7 and $w=30$. As we can notice the conductance curves are practically the same except for the vertical offset between them, see Fig. 3a,b. By following our protocol and using the auxiliary conductance-related quantity we can derive a general expression that connects the conductance patterns of complex structures with different barrier heights, namely:

14 $$\begin{aligned} {\mathbb {G}}^{\sigma }_{\Delta }(E_{F})\approx \frac{\left[ {\mathbb {G}}^{\sigma }_{\frac{\Delta }{2}}(E_{F})\right] ^{\beta _{\sigma }}}{(2)^{\beta _{\sigma }-1}}, \end{aligned}$$

where $\beta _{\sigma }$ is a non-constant exponent that depends on the structural parameters as well as the spin component. For the cases presented in Fig. 3 the exponents take values $\beta _{+1}=2.87$ and $\beta _{-1}=3.02$. As we can see in Fig. 3c,d the scaling is fairly good for both SU and SD. Here, the scaled curve corresponds to $\Delta =1$, while the reference one to $\Delta =2$. It is important to mention that the fundamental differences between the scaled and reference curves take place in the low-energy side, being more important for SD.

**Figure 14:**
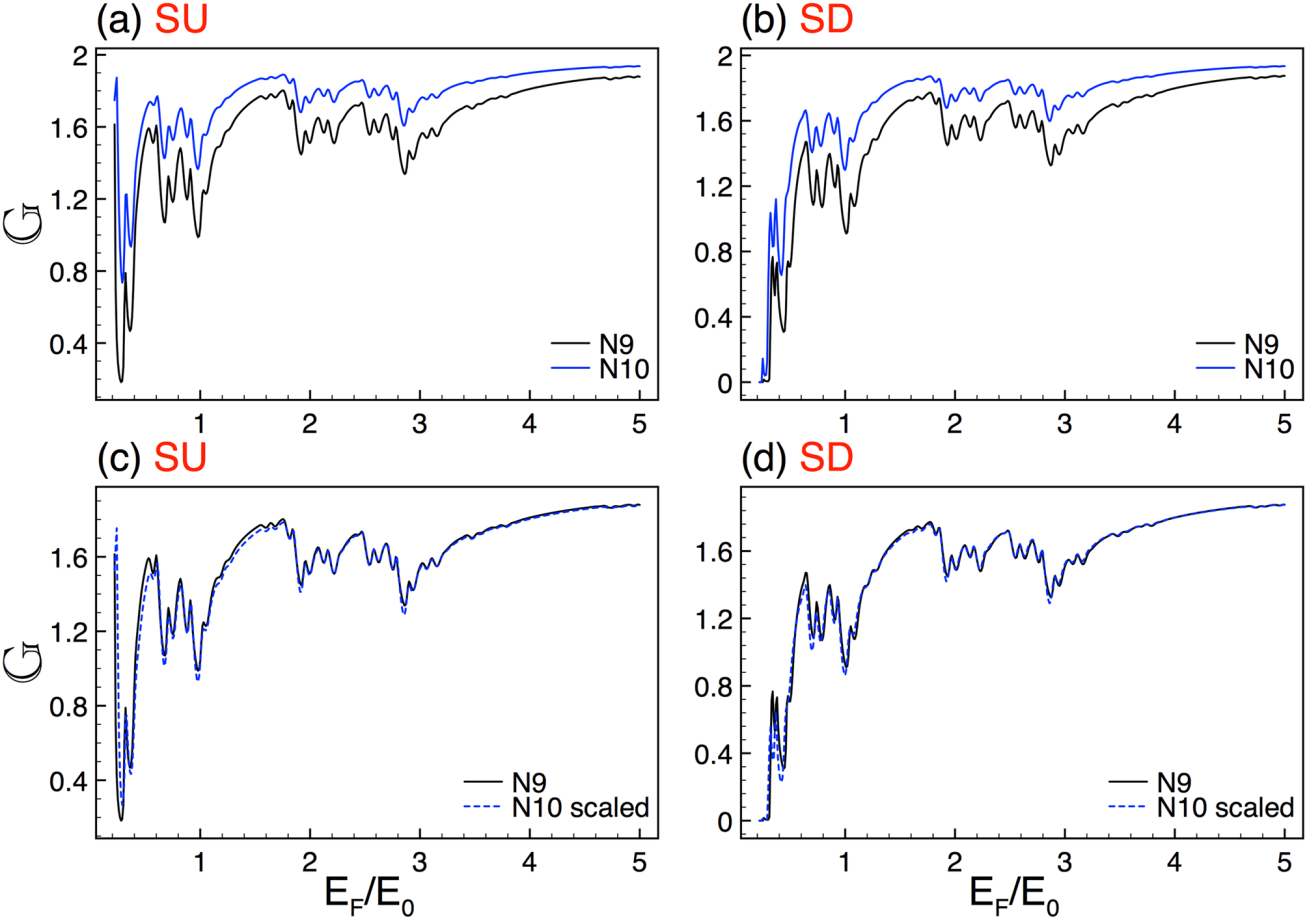
The same as Fig. 13, but here the generations are *N*9 and *N*10, reference and scaled curve, respectively. In this case the scale factors take values $\alpha _{+1}=2.01$ and $\alpha _{-1}=1.95$.

**Table 1:** Evolution of the scaling exponents for different generations.

Scaling exponents		
Generations	SU ($\alpha _{+}$)	SD ($\alpha _{-}$)
*N*6–*N*7	1.78	1.83
*N*7–*N*8	1.83	1.89
*N*8–*N*9	1.87	1.86
*N*9–*N*10	2.01	1.95

In general, the structural parameters ($N, \Delta , w$) and the spin components determine the value of the scaling exponents. For *N*8–*N*9 and *N*9–*N*10 we adjust the length of the system in order to have reasonable barrier widths and not approaching to the interatomic distance in silicene. In particular, we have used $w=45$, instead of $w=30$ considered for *N*6–*N*7 and *N*7–*N*8.

**Figure 15:**
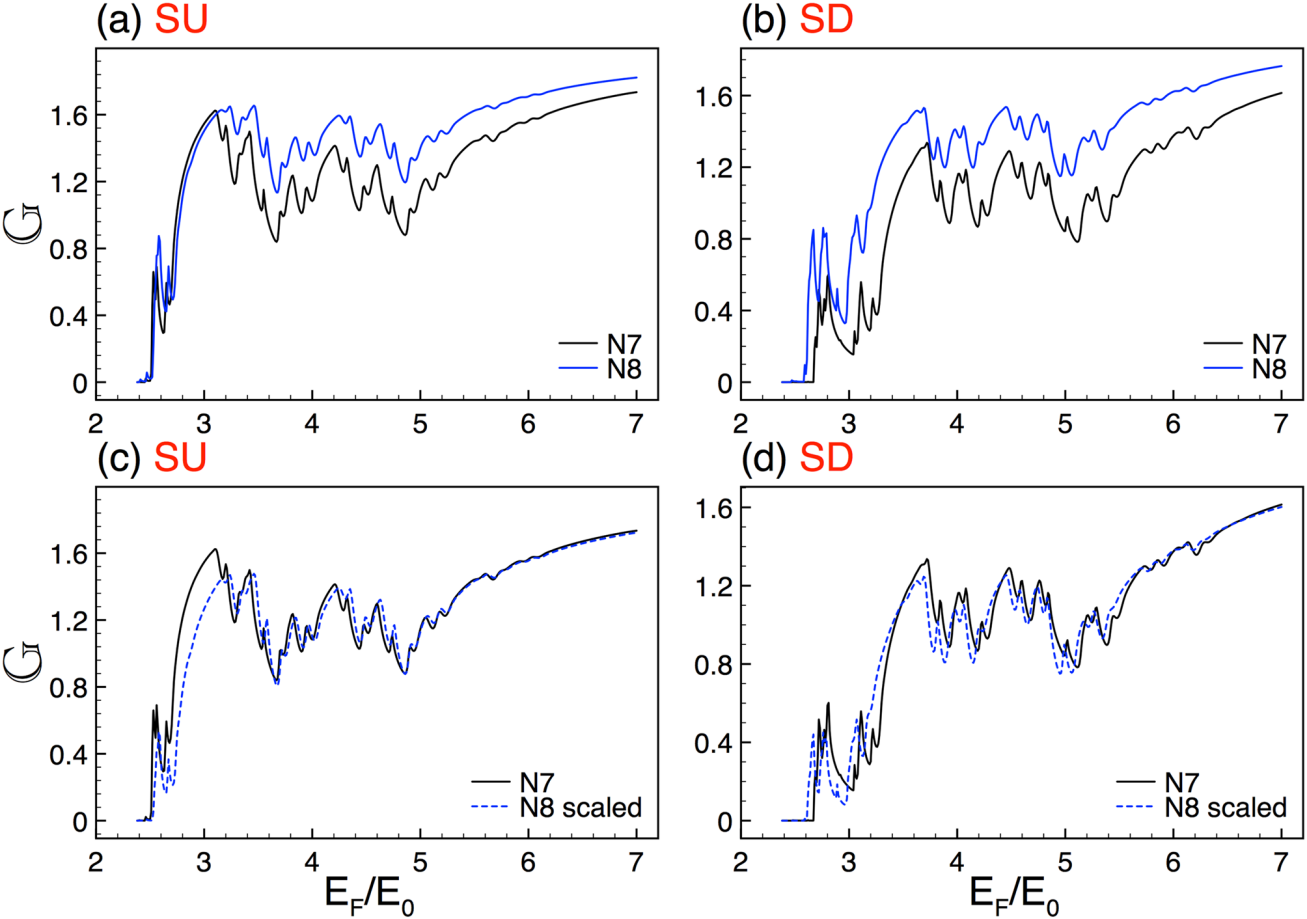
Self-similar conductance patterns in complex germanene structures. The SOC in germanene is of the order of 43 meV. The panel distribution is the same as in Fig. 2, but here the scale factors are $\alpha _{+1}=1.60$ and $\alpha _{-1}=1.77$.

Regarding the length of the system, we can also obtain self-similar conductance patterns for complex structures with different lengths. In Fig. 4 we show the conductance versus de Fermi energy for systems with lengths $w=20$ and $w=10$. The generation and height of the barriers are *N*7 and $\Delta =2$. Figure 4a,b illustrate the corresponding conductance patterns for SU and SD, respectively. Our results, at first glance, indicate that these patterns cannot be directly connected. In fact, the curves envelopes are not at all similar. However, by contracting the energy axis and choosing an appropriate exponent it is possible to connect the conductance patterns, see Fig. 4c,d. The general expression for this scaling is given as

15 $$\begin{aligned} {\mathbb {G}}^{\sigma }_{w}(E_{F})\approx \frac{\left[ {\mathbb {G}}^{\sigma }_{\frac{w}{2}}(\frac{E_{F}}{2})\right] ^{\gamma _{\sigma }}}{(2)^{\gamma _{\sigma }-1}}, \end{aligned}$$

with $\gamma _{\sigma }$ the scaling exponent. For the cases of Fig. 4 the exponents are $\gamma _{+1}=\gamma _{-1}=3.2$. As we can see in Fig. 4c a good matching for SU is obtained, except for $E_F<0.4E_0$. For SD we obtain similar results. However, the coincidence between the scaled and reference curve is substantially better for $E_F>1.2E_0$, see the dashed-blue curve in Fig. 4d. Here, it is important to remark that the exponents for both spin components are the same. However, in general, they depend on the structural parameters and the spin component.

**Figure 16:**
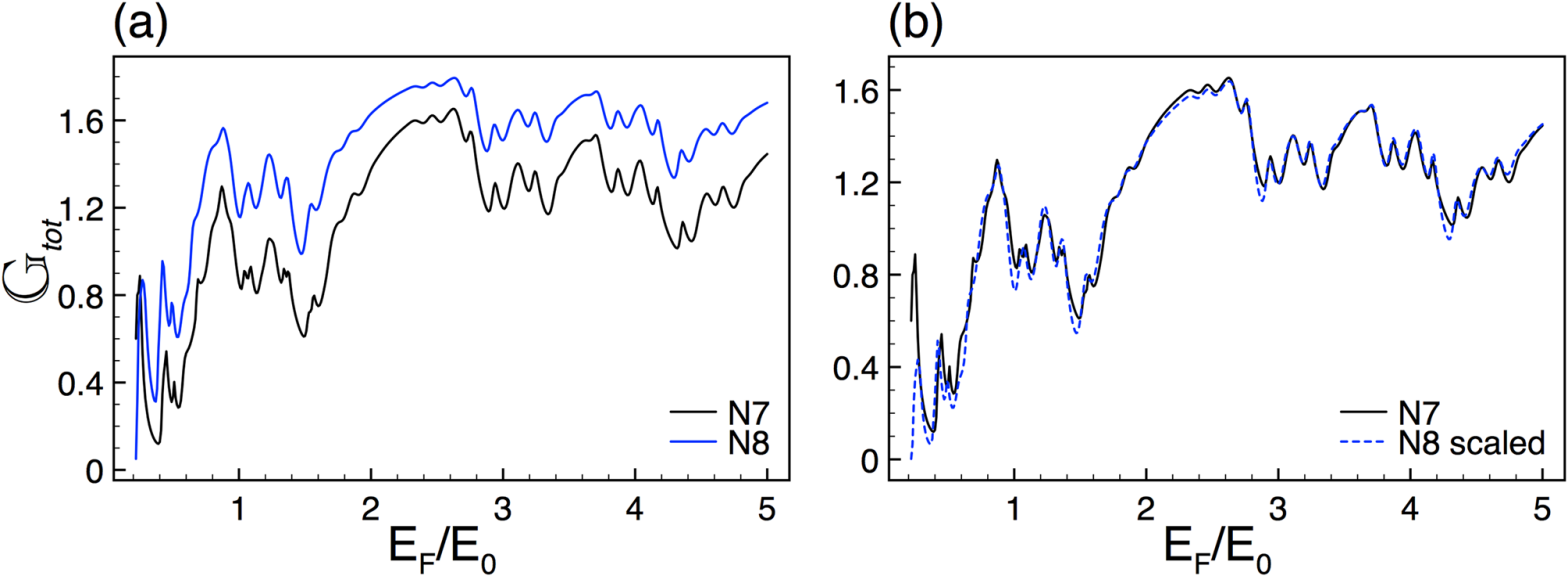
The same as Fig. 2, but now for the total conductance. In the present case the scale factor is $\alpha =1.84$.

**Figure 17:**
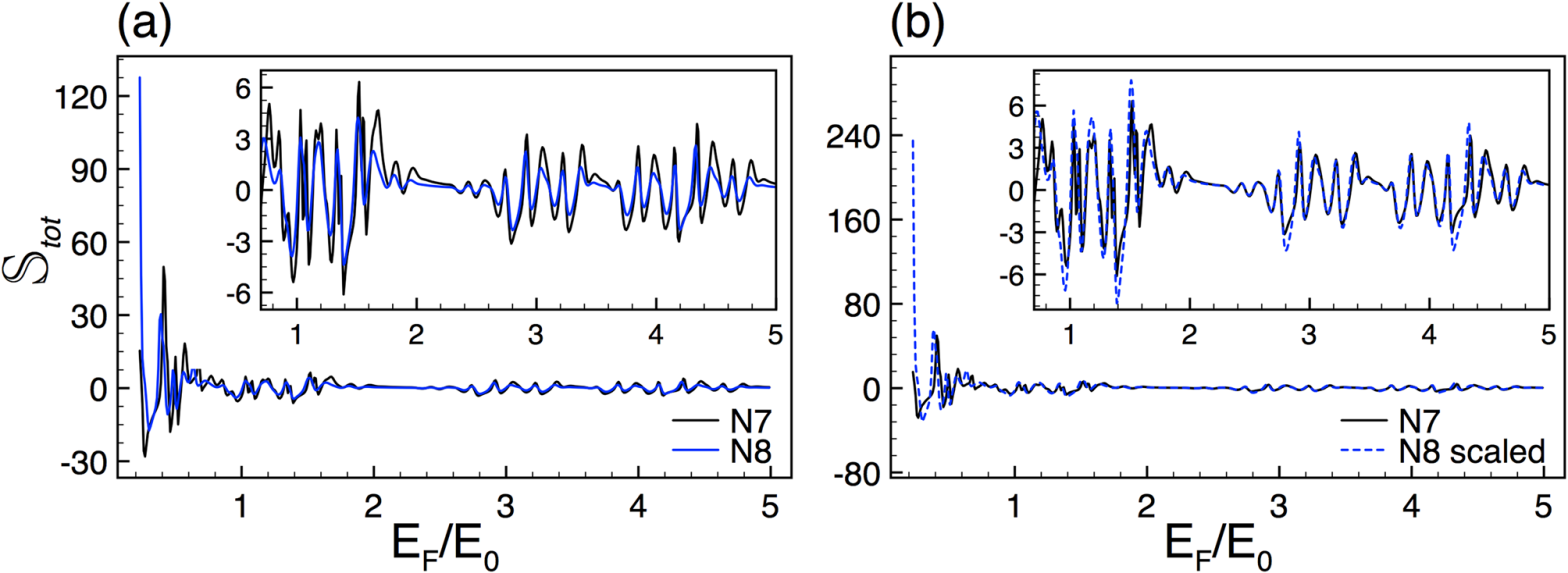
The same as Fig. 8, but now for the total Seebeck coefficient. The scale factor is the same as in Fig. 16. The average temperature considered is 50 K.

We consider that the present results are remarkable because in principle conductance-related quantities such as the spin polarization and the Seebeck coefficient could display self-similar characteristics as well. Moreover, even though we know the scaling rules for the conductance patterns there is no guarantee that the scaling for the spin polarization and the Seebeck coefficient can be obtained following the conductance protocol and that the scale factors be directly related to the conductance ones.

### Spin polarization

In second place, we analyze the results of the so-called conductance spin polarization $P_{C}$. By using Eq. (11) it is possible to calculate $P_{C}$ as a function of the Fermi energy for different *N*, $\Delta$ and *w*. The corresponding spin polarization curves also present self-similar characteristics. However, in the present case, there is no an auxiliary quantity that helps us to unveil the scaling rules. So, we proceed numerically by testing different scale factors in order to connect the self-similar patterns. To illustrate our scaling results we use the same generations, heights of the barriers and lengths of the system as in the conductance case.

In Fig. 5a,b we show the conductance spin polarization patterns for generations *N*7 and *N*8, the other structural parameters are the same as in Fig. 2. As we can notice the spin polarization spectra look quite similar, to be specific, we can see similar curve envelopes for energies greater than $E_{0}$. A better perspective of the self-similar characteristics is presented in the subview. Here, it is important to remark that the spin polarization can take both positive and negative values, as a consequence, a dilatation transformation is implemented instead of an exponent as in the conductance rules. With these considerations, the general expression that connects the self-similar spin polarization patterns between two generations is given as

16 $$\begin{aligned} P_{C,N}(E_{F})\approx 2\left[ P_{C,N+1}(E_{F})\right] , \end{aligned}$$

resulting in a simpler relation than in the conductance case. A comparison between the reference and scaled pattern (dashed-blue lines) is illustrated in the subview of Fig. 5b. As we can notice a good matching is obtained.

The spin polarization curves for different heights in the barriers also display self-similar characteristics. In Fig. 6a we show the spin polarization results for $\Delta =2$ and $\Delta =1$. We have considered the same structural parameters as in Fig. 3. As we can see the curves are not at all similar at low energies. However, as the energy increases the resemblance between the spin polarization patterns improves, see the subview in Fig. 6a. By testing different scale factors, it is possible to go from one curve to the other with the following expression

17 $$\begin{aligned} P_{C,\Delta }(E_{F})\approx 4\left[ P_{C,\frac{1}{2} \Delta }(E_{F})\right] . \end{aligned}$$

In this case, the scale factor is twice the one found for the scaling between generations. In the subview of Fig. 6b we show the concrete results of applying Eq. (17). In some energy intervals the scaling works reasonable well, while in others, mainly at low energies, the coincidence between the scaled and reference curve is far from good.

Systems with different lengths also manifest self-similar spin polarization patterns. The results for $w=20$ and $w=10$ are shown in Fig. 7a. The generation and the height of the barriers are the same as in Fig. 4. As in the conductance case, at first instance, there are no self-similar characteristics. So, in order to connect the spin polarization curves it is necessary to scale the Fermi energy as well as to choose appropriately the proportional factor between the curves. In specific, the scaling relation is given as

18 $$\begin{aligned} P_{C,w}(E_{F})\approx 8\left[ P_{C,\frac{1}{2} w} \left( \frac{E_{F}}{2} \right) \right] . \end{aligned}$$

Here, the scale factor is twice the one for the scaling between heights. The results of the scaling according to Eq. (18) are shown in Fig. 7b. In the subview we can see more details about the matching between the scaled and reference curves. As in the other cases, the scaling improves as the Fermi energy increases.

These results are quite interesting because despite $P_{C}$ is an intricate average of the conductance spin components the self-similar characteristics prevail for all the structural parameters. Furthermore, the three scaling rules proposed for the spin polarization work reasonably well as the Fermi energy increases. It is worth mentioning that we obtain similar results for the so-called tunneling spin polarization $P_T$, which is equivalent to $P_{C}$ but defined in terms of the transmittance. The details can be found in the Supplementary information.

### Thermoelectric effects

Finally, we study the thermoelectricity for the electron spin components due to the hot and cold contacts that generate a temperature gradient along the complex silicene structures. In particular, we have focused our attention in the well-known Seebeck coefficient. To compute it, we first have to redefine it as ${\mathbb {S}}^{\sigma }(E_F)=S^{\sigma }(E_F)/S_{0}$, with $S_{0}=\pi ^{2} k_{\beta }^{2} T/3e$ the fundamental thermopower unit. In order to be consistent, we used the same structural parameters than the ones for the conductance and the spin polarization.

Regarding generations, Fig. 8 shows the Seebeck coefficient results for *N*7 and *N*8. Fig. 8a,b correspond to SU and SD, respectively. As we can notice the Seebeck coefficient curves are remarkably similar, see the subviews. Even, the resemblance is superior to the one found for the spin polarization curves. So, once we are sure that this quantity reflects evidence of self-similarity, the matter now is limited to deal with the scalability. As we have corroborated with the spin polarization there is no a route or procedure to derive the scaling relations. In the present case, we take advantage of the direct relation between the Seebeck coefficient and the conductance in order to obtain the scaling expressions. In particular, we use the definition of the Seebeck coefficient for an arbitrary generation *N*

19 $$\begin{aligned} \left. {\mathbb {S}}^{\sigma }_{N}(E) = S_{0}\dfrac{\partial \ln \left[ {\mathbb {G}}^{{\sigma }}_{N}(E)\right] }{\partial E} \right| _{E=E_F}, \end{aligned}$$

by substituting the scaling relation for ${\mathbb {G}}^{{\sigma }}_{N}(E)$ (Eq. 13)

20 $$\begin{aligned} \left. {\mathbb {S}}^{\sigma }_{N}(E) \approx S_{0}\dfrac{\partial \ln \left[ \frac{[{\mathbb {G}}^{\sigma }_{N+1}(E)]^{\alpha _{\sigma }}}{(2)^{\alpha _{\sigma }-1}}\right] }{\partial E} \right| _{E=E_F}, \end{aligned}$$

and using the properties of the logarithmic function we can obtain the expression that connects the Seebeck coefficient between generations

21 $$\begin{aligned} {\mathbb {S}}^{\sigma }_{N}(E_F)\approx \alpha _{\sigma }\left[ {\mathbb {S}}^{\sigma }_{N+1}(E_F)\right] . \end{aligned}$$

This expression is surprisingly simple, mathematically equivalent to the scaling relations found for the spin polarization. However, the scaling is much better for the Seebeck coefficient. In Fig. 8c,d we show how Eq. (21) works for SU and SD. As compare with the spin polarization scaling (see Fig. 5), the matching between the scaled and reference Seebeck curves is fairly good for both spin components, being superior for SU. It is also interesting to note that the scale factors are the same for both the conductance and the Seebeck coefficient, however for the latter the scale factors come in multiplicative fashion.

The Seebeck coefficient of complex structures with different barrier heights also display self-similar characteristics. In Fig. 9, we show the Seebeck coefficient results for $\Delta =2$ (solid-black lines) and $\Delta =1$ (solid-blue lines). Figure 9a,b correspond to SU and SD, respectively. To obtain the scaling relations we follow the previous analytic procedure. In specific, the scaling relation of the Seebeck coefficient for complex structures with different barrier heights is given as

22 $$\begin{aligned} {\mathbb {S}}^{\sigma }_{\Delta }(E_F)\approx \beta _{\sigma }\left[ {\mathbb {S}}^{\sigma }_{\frac{1}{2} \Delta }(E_F)\right] , \end{aligned}$$

where $\beta _{\sigma }$ is the same exponent that connects ${\mathbb {G}}^{\sigma }_{\Delta }$ and ${\mathbb {G}}^{\sigma }_{\frac{1}{2} \Delta }$. The results of the scaling for SU and SD are shown in Fig. 9c,d, respectively. As in the conductance case (Fig. 3) the factors adopt the values $\beta _{+1}=2.87$ and $\beta _{-1}=3.02$. As we can see in the subviews of Fig. 9c,d, a good coincidence between the scaled and reference curves is obtained.

In the last place, we explore the Seebeck coefficient results of complex structures with different lengths. In Fig.  10 we show the corresponding results for $w=20$ (solid-black lines) and $w=10$ (solid-blue lines). Figure 10a,b correspond to SU and SD, respectively. The generation and the height of the barriers are the same as in Fig. 4. As in the previous cases, the self-similar Seebeck coefficient patterns can be connected with an appropriate scaling relation. By using the definition of the Seebeck coefficient (Eq. 12) and the scaling rule of the conductance between lengths (Eq. 15) we can obtain the corresponding scaling relation for the Seebeck coefficient

23 $$\begin{aligned} {\mathbb {S}}^{\sigma }_{w}(E_F)\approx \gamma _{\sigma }\left[ {\mathbb {S}}^{\sigma }_{\frac{1}{2}w}\left( \frac{E_{F}}{2}\right) \right] , \end{aligned}$$

with $\gamma _{\sigma }$ the same exponent that connects ${\mathbb {G}}^{\sigma }_{w}$ and ${\mathbb {G}}^{\sigma }_{\frac{1}{2}w}$. The results of the scaling for SU and SD are shown in Fig. 10c,d, respectively. The scale factors take values of $\gamma _{+}=\gamma _{-}=3.2$, which are the same as in the conductance case, see Fig. 4. As we can appreciate in the subviews the scaling works quite well, being better for SU. In this case, initially, the curves are far from similar, but once the energy axis is contracted and the Seebeck coefficient multiply by the correct factor, we recover in high degree the reference curve.

We consider that the present results are quite interesting because as far as we know it is the first time that a conductance related quantity like the Seebeck coefficient presents self-similar characteristics. Moreover, that we can obtain the scaling rules straightforwardly through the analytic relation between the conductance and Seebeck coefficient.

## Discussion

The first thing that we want to put on the table it is the scaling rules. As we have mentioned, scaling rules are tricky. In fact, despite we can see self-similar patterns in a particular physical property it is not straightforward to obtain the scaling relations. According to our experience it is better to deal with probabilistic quantities, which give us the possibility to test different scale factors to connect the self-similar patterns. For instance, a quantity that exemplify this it is the transmission probability or transmittance. In the Supplementary information we show the self-similar patterns and the scaling results for this quantity. Knowing the scaling rules for the transmittance we can think that the conductance scaling is obtainable via the direct relation between the conductance and transmittance. However, it is not the case, by substituting the scaling expressions of the transmittance in Eq. (10) we cannot derive analytically the scaling relations for the conductance. Fortunately, we can define a probabilistic auxiliary quantity that help us to obtain the conductance scaling rules. By comparing the scaling expressions for the transmittance and conductance we can realize that they are similar, but in the conductance case we have additional intricate factors that make really challenging the derivation of the scaling rules without the assistance of the auxiliary quantity.

Other aspect that we want to address is the one related to the generation at which the self-similar characteristics arise. Actually, the first generations of the complex structure are far to be self-similar and consequently it is not expected that the physical properties manifest self-similar characteristics. According to our calculations and the system parameters that we are considering the self-similar characteristics take place at the sixth generation. In Fig. 11 we show the results of the linear-regime conductance for generations *N*5 and *N*6. As we can see the conductance patterns look quite similar. However, by scaling *N*6 according to Eq. (13) we obtain that the matching between the scaled and reference curve is not as good as the corresponding one between *N*6 and *N*7, see Fig. 12. If we consider lower generations the scaling is even worst, results not shown. To know the generation at which the self-similar characteristics are taking place is fundamental for experimentalists as well as for possible applications. In fact, it is the equivalent to know the minimum periods required to see the fundamental characteristics of semiconductor superlattices, which in general it is established as ten periods.

In relation to scaling, we repeatedly seen that it improves as the Fermi energy increases. Actually, there is no a fundamental explanation about it. However, it is plausible that the differences between the scaled and reference structure be less perceptible as we are far from the energy region in which the perturbation (self-similar potential) is preponderant, giving rise to a better scaling. We also testified that scaling improves as generation grows. This is expected because the complex structures will approach to true self-similar objects for larger generations. In fact, the scaling improvement covers a wider energy range, including the low-energy side, see the results for generations N8, N9 and N10 in Figs. 13 and 14. Furthermore, we can notice that the scaling exponents vary gradually as generation grows, tending (both spin components) to a value around 2. The specific exponents can be seen in Table 1. It is quite interesting that the exponents tend to the value found for the scaling of the transmittance. The details can be found in the Supplementary information.

Regarding the energy range at which the self-similar characteristics are taking place in principle it is accessible from the experimental standpoint^42,43^. However, if for a particular application the energy range required is higher, silicene is not necessarily an option. In addition, the spin-resolved transport is lost as the Fermi energy increases. To expand the energy range without compromising the spin-resolved properties we can opt for the silicene’s twin material germanene^44–46^. In fact, the SOC in germanene is more than ten times larger than in silicene^24,25^. In Fig. 15 the conductance scaling results for complex germanene structures are shown. As we can notice the self-similar conductance patterns are presented regardless of the energy range in question.

Finally, it is worth mentioning that in order to have spin polarization response in our system a valley-polarized filter is required. By considering the definition of conductance spin polarization

24 $$\begin{aligned} P_C=\dfrac{\sum _{\eta }G^{\eta ,+} - G^{\eta ,-}}{\sum _{\eta }G^{\eta ,+} + G^{\eta ,-}}, \end{aligned}$$

we can see that the spin polarization is zero due to the reversing of the spin components in the $K'$ valley. For valley polarization we also need a spin-polarized filter, otherwise the same null result as in the conductance spin polarization is obtained. As an alternative we can use ferromagnetic electrodes to generate effective valley and spin polarization in silicene^47–49^.

In the case of the total conductance

25 $$\begin{aligned} G_{tot}=\frac{G^{K,+}+G^{K,-}+G^{K',+}+G^{K',-}}{4}=\frac{G^{K,+}+G^{K,-}}{2}, \end{aligned}$$

and the total Seebeck coefficient

26 $$\begin{aligned} \left. S_{tot}(E)=\dfrac{\pi ^{2} k_{\beta }^{2} T}{3e}\dfrac{\partial \ln [ G_{tot}(E)]}{\partial E} \right| _{E=E_F}, \end{aligned}$$

the self-similar patterns prevail, see Figs. 16 and 17. We consider that these results are quite relevant because in principle we can see the self-similar phenomenon in the conductance and Seebeck coefficient without the need of a spin-polarized filter.

## Conclusions

In summary, we study the transport, spin polarization and thermoelectric effects in Cantor-like silicene structures. The theoretical treatment is based on the transfer matrix approach, the Landauer–Büttiker formalism and the Cutler–Mott formula. We demonstrate that when silicene is nanostructured in complex fashion, it reflects self-similarity in the physical properties. In particular, we find self-similar patterns at different scales in the conductance, the spin polarization and the Seebeck coefficient for both electron spin components. Furthermore, the self-similar patterns of the mentioned physical quantities are well characterized by concrete scaling rules. The structural parameters of the system are directly involved in the scaling expressions: the generation of the structure, the height of the barriers and the length of the system. We consider that our findings are not only important to understand the behavior of charge carriers in complex structures, they could also be relevant (useful) in spintronic and thermoelectric applications due to the scalability of the spin polarization and the Seebeck coefficient. To our knowledge, this is the first time that a 2D material beyond monolayer graphene presents self-similar transport. Moreover, a material that manifests self-similar characteristics in relevant quantities such as the spin polarization and the Seebeck coefficient.

## Supplementary information

Supplementary Information.
