# Supplementary material for: Self-similar transport, spin polarization and thermoelectricity in complex silicene structures
Source: Sci Rep. 2020 Sep 7;10:14679. doi: 10.1038/s41598-020-71697-1 (PMC7477273; doi:10.1038/s41598-020-71697-1)
Supplement: Supplementary file 1 — Supplementary Information. [file 41598_2020_71697_MOESM1_ESM.pdf]

Supplementary Information

Self-similar transport, spin polarization and thermoelectricity in complex silicene structures

R. Rodríguez-González[1], L. M. Gaggero-Sager[1] and I. Rodríguez-Vargas[2]

[1] *Centro de Investigación en Ingeniería y Ciencias Aplicadas, Universidad Autónoma del Estado de Morelos, Av. Universidad 1001, Col. Chamilpa, 62209, Cuernavaca, Morelos, México.*

[2] *Unidad Académica de Ciencia y Tecnología de la Luz y la Materia, Universidad Autónoma de Zacatecas, Carretera Zacatecas-Guadalajara Km. 6, Ejido La Escondida, 98160 Zacatecas, Zac., México.*

In this supplementary material, we present the scaling rules for the transmission, tunneling spin polarization and conductance-related quantity $\mathbb{H}$. In particular, scaling expressions for the three fundamental structural parameters: the generation number N, the height of the barriers Δ and the length of the system w. All numerical calculations correspond to the K valley. So, the valley index η is omitted in all expressions.

S1. Scaling rules for the transmission properties

The transmittance between generations scales as,

$$T_N^\sigma(E) \approx \left[T_{N+m}^\sigma(E)\right]^{2^m}, \tag{S1.1}$$

where m is the difference between two non-consecutive generations and $\sigma = \pm 1$ stands for the electron spin components. Concrete results for this scaling are shown in Fig. S1.

The transmittance for different barrier heights scales as,

$$T^{\sigma}_{\Delta}(E) \approx \left[T^{\sigma}_{\frac{1}{k}\Delta}(E)\right]^{k^2}, \tag{S1.2}$$

where k is the factor that relates the heights of the barrier. Specific results for this scaling are shown in Fig. S2.

The transmittance for different lengths of the system can be connected with the following expression,

$$T^{\sigma}_{w}(E) \approx \left[T^{\sigma}_{\frac{1}{\alpha}w}(\frac{1}{\alpha}E)\right]^{\alpha^2}, \tag{S1.3}$$

where α is the factor that relates the lengths of the system. Scaling results for this case can be appreciated in Fig. S3.

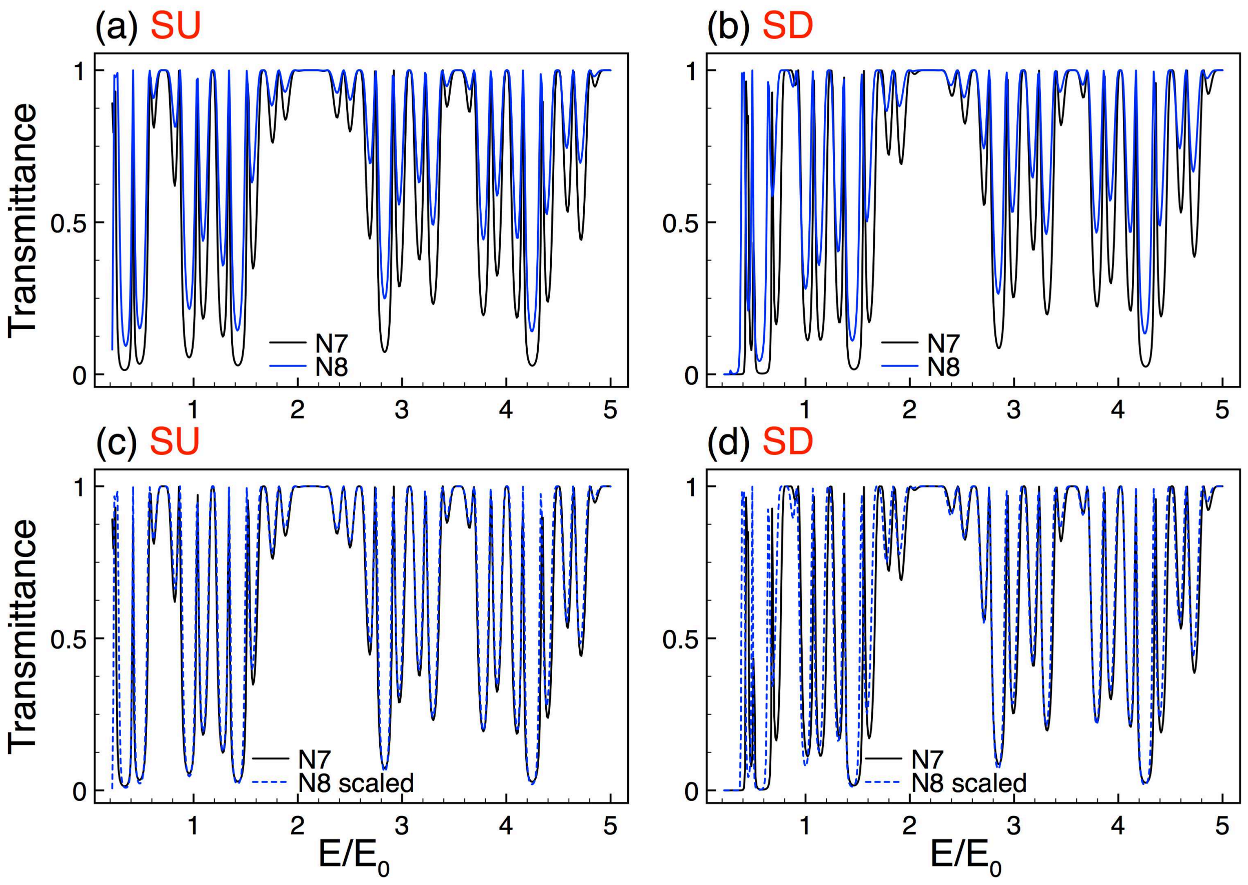

Fig. S1: Transmittance scaling between generations for the spin up (SU) and spin down (SD) component. (a) and (b) represent the transmittance curves for the seventh $N7$ (solid-black lines) and eighth $N8$ (solid-blue lines) generations. (c) and (d) are the same as in (a) and (b), but here $N8$ (dashed-blue lines) is transformed according to Eq. (S1.1). The other structural parameters are $\Delta = 2$ and $w = 30$.

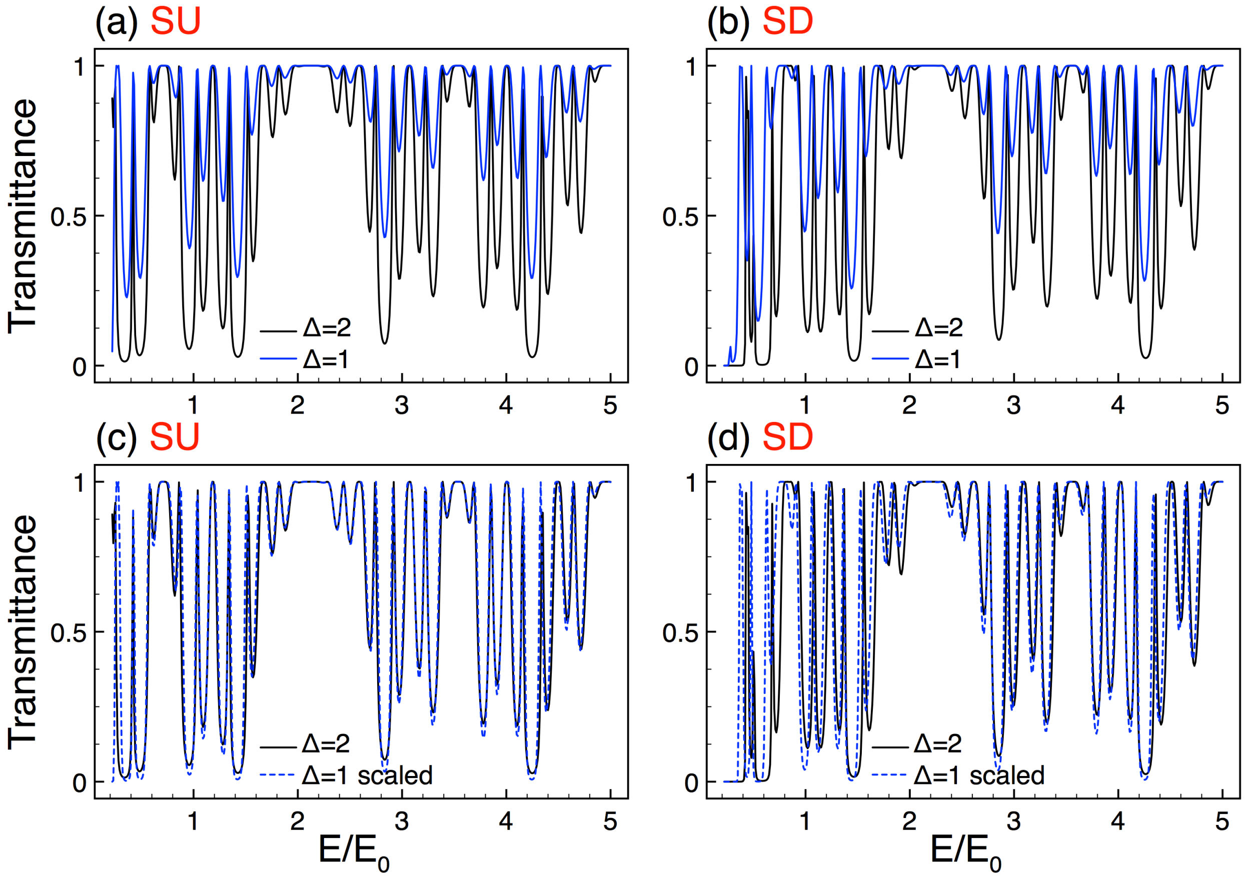

Fig. S2: Transmittance scaling between barrier heights for SU and SD. (a) and (b) illustrate the transmittance curves for $\Delta = 2$ (solid-black lines) and $\Delta = 1$ (solid-blue lines). (c) and (d) are the same as in (a) and (b), but now the spectrum of $\Delta = 1$ (dashed-blue lines) is scaled with Eq. (S1.2). The other structural parameters are $N7$ and $w = 30$.

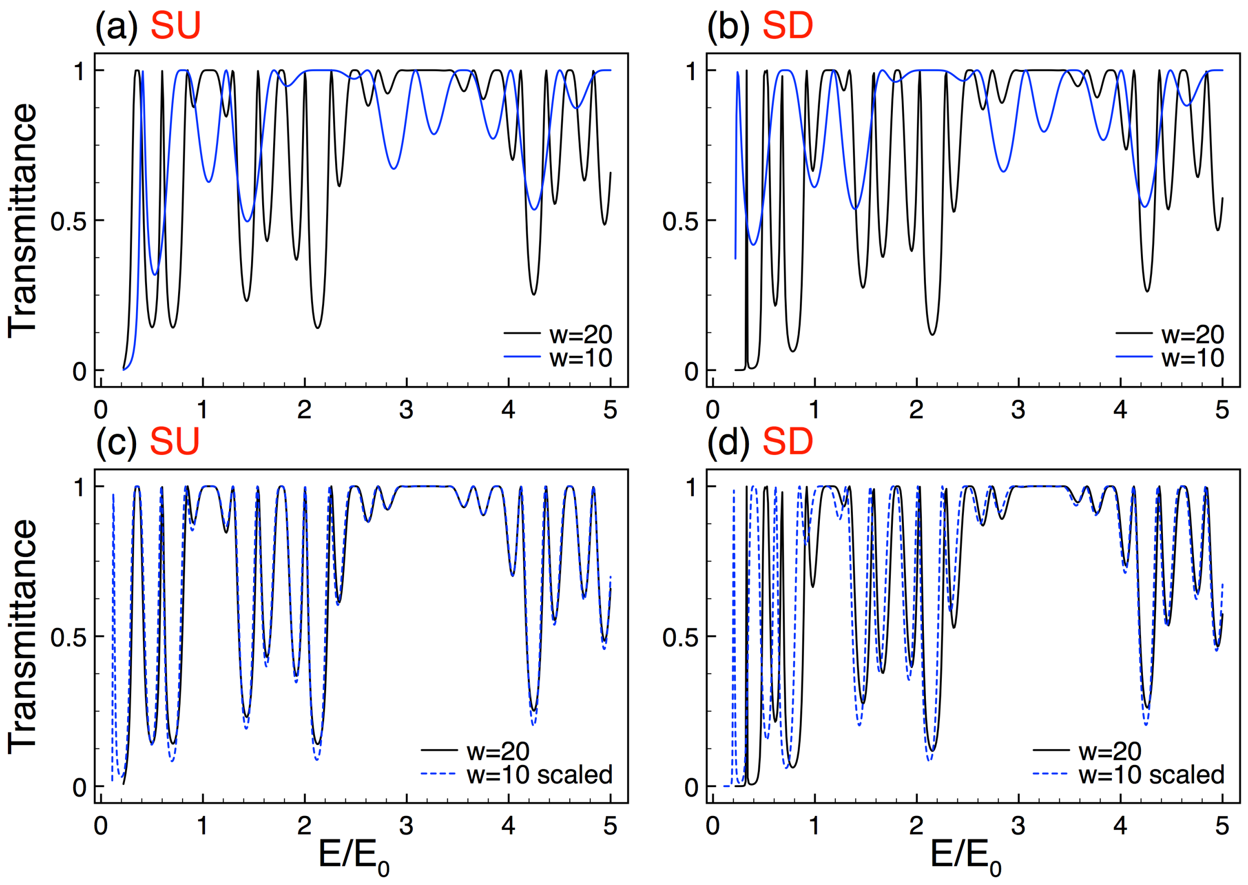

Fig. S3: Transmittance scaling between lengths for SU and SD. (a) and (b) stand for the transmittance curves for $w = 20$ (solid-black lines) and $w = 10$ (solid-blue lines). (c) and (d) are the same as in (a) and (b), but the curve for $w = 10$ (dashed-blue lines) is scaled applying Eq. (S1.3). The other structural parameters are $N7$ and $\Delta = 2$.

S2. Scaling rules for the tunneling spin polarization

Another physical property that reveals self-similar behaviour in complex silicene structures is the so-called tunneling spin polarization P_T. This quantity is defined equivalently as the conductance spin polarization, namely:

$$P_T = \frac{T^+ - T^-}{T^+ - T^-}. \tag{S2.1}$$

The corresponding scaling expression between generations is given as:

$$P_{T,N}(E) \approx 2[P_{T,N+1}(E)], \tag{S2.2}$$

here a multiplication factor is used because a dilatation transformation is needed. A specific case is illustrated in Fig. S4.

The spin polarization for different barrier heights scales as

$$P_{T,\Delta}(E) \approx 4\left[P_{T,\frac{1}{2}\Delta}(E)\right]. \tag{S2.3}$$

A particular case is shown in Fig. S5.

In the case of different lengths of the system the scaling comes as

$$P_{T,w}(E) \approx 10\left[P_{T,\frac{1}{2}w}\left(\frac{E}{2}\right)\right], \tag{S2.4}$$

here a contraction in the energy axis for the scaled curve is required. One can see how this equation works in Fig. S6.

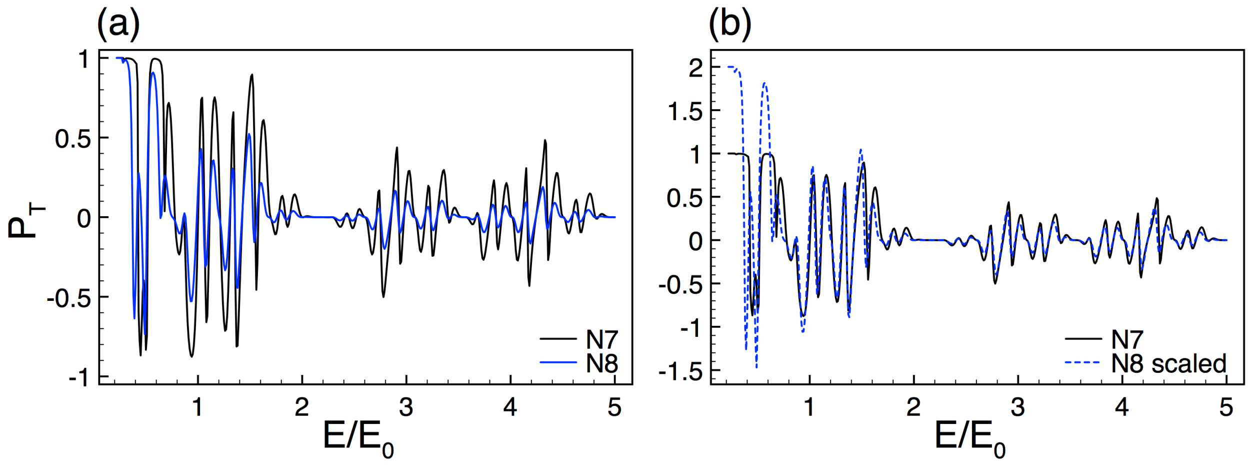

Fig. S4: Tunneling spin polarization scaling between generations. (a) Tunneling polarization curves for the seventh $N7$ (solid-black lines) and eighth $N8$ (solid-blue lines) generations. (b) The same as in (a), but here Eq. (S2.2) is used to scaled generation $N8$ (dashed-blue lines). The rest of the structural parameters are the same as in Fig. S1.

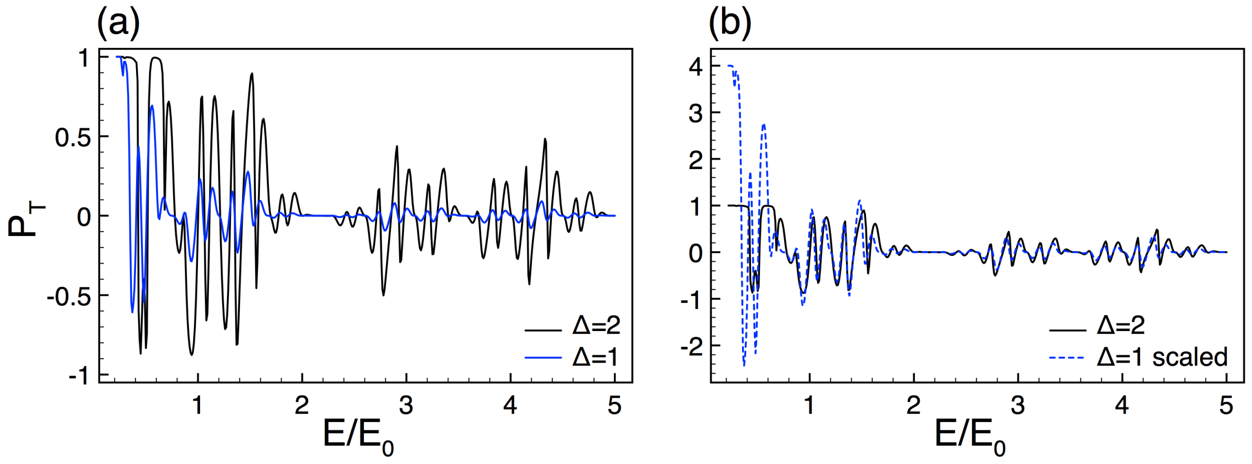

Fig. S5: Tunneling spin polarization scaling between barrier heights. (a) Tunneling spin polarization curves for $\Delta = 2$ (solid-black lines) and $\Delta = 1$ (solid-blue lines). (b) The same as in (a), but here the curve for $\Delta = 1$ (dashed-blue lines) is transformed according to Eq. (S2.3). The rest of the structural parameters are the same as in Fig. S2.

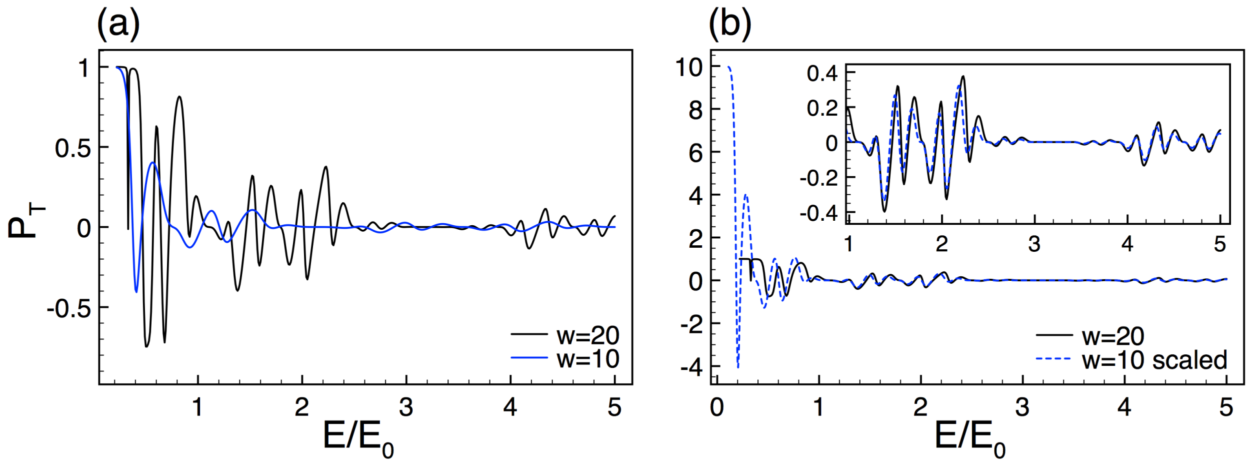

Fig. S6: Tunneling spin polarization scaling between lengths. (a) Tunneling spin polarization curves for $w = 20$ (solid-black lines) and $w = 10$ (solid-blue lines). (b) The same as in (a), but here the length $w = 10$ (dashed-blue lines) is scaled according to Eq. (S2.4). The subview in (b) illustrates more clearly the scalability. The rest of the structural parameters are the same as in Fig. S3.

S3. Scaling rules for the conductance-related quantity $\mathbb{H}$

To find out the scaling rules in the conductance, firstly it is necessary to implement an auxiliary quantity denoted by the symbol $\mathbb{H}$ and valid between 0 and 1, which can be written as,

$$\mathbb{H}^{\sigma} = \frac{H^{\sigma}}{H_0} = \frac{1}{2}\int_{-\pi/2}^{\pi/2} T^{\sigma}(E_F, \theta)\cos\theta d\theta, \tag{S3.1}$$

here σ indicates the spin index, E_F is the system Fermi energy, $H_0 = e^2 L_y E_0/2h^2 v_F$ is the fundamental $\mathbb{H}$-conductance factor, with L_y begin the size of the system along the transverse y-direction and θ represents the incident angle of the impinging electrons with respect to the propagation x-direction.

With this information at hand, it is possible to propose scaling expressions for the auxiliary quantity $\mathbb{H}$ for the fundamental structural parameters N, Δ and w.

In the case of generations the scaling is given as

$$\mathbb{H}^{\sigma}_{N}(E_F) \approx \left[\mathbb{H}^{\sigma}_{N+1}(E_F)\right]^{\alpha_\sigma}, \tag{S3.2}$$

where α_σ is an exponent that depends of the structural parameters of the system as well as of the spin index. In Fig. S7 we can see this type of scalability.

Regarding the height of the barriers the scaling is

$$\mathbb{H}^{\sigma}_{\Delta}(E_F) \approx \left[\mathbb{H}^{\sigma}_{\frac{1}{2}\Delta}(E_F)\right]^{\beta_\sigma}. \tag{S3.3}$$

As in the case of generations the exponent β_σ depends of the structural parameters and the spin component. A specific case is illustrated in Fig. S8.

For lengths of the system, we have

$$\mathbb{H}^{\sigma}_{w}(E_F) \approx \left[\mathbb{H}^{\sigma}_{\frac{w}{2}}(\frac{E_F}{2})\right]^{\gamma_\sigma}, \tag{S3.4}$$

with γ_σ a structural and spin dependent exponent. Fig. S9 shows how this scaling works.

Once we obtain the scaling expressions we relate $\mathbb{H}$ with the conductance $\mathbb{G}$ as follows

$$\mathbb{H}^{\sigma} = \frac{1}{2E_F}\mathbb{G}^{\sigma}, \tag{S3.5}$$

where $\mathbb{G} = G/G_0$ and G_0 is given in terms of H_0 as $G_0 = 2H_0$.

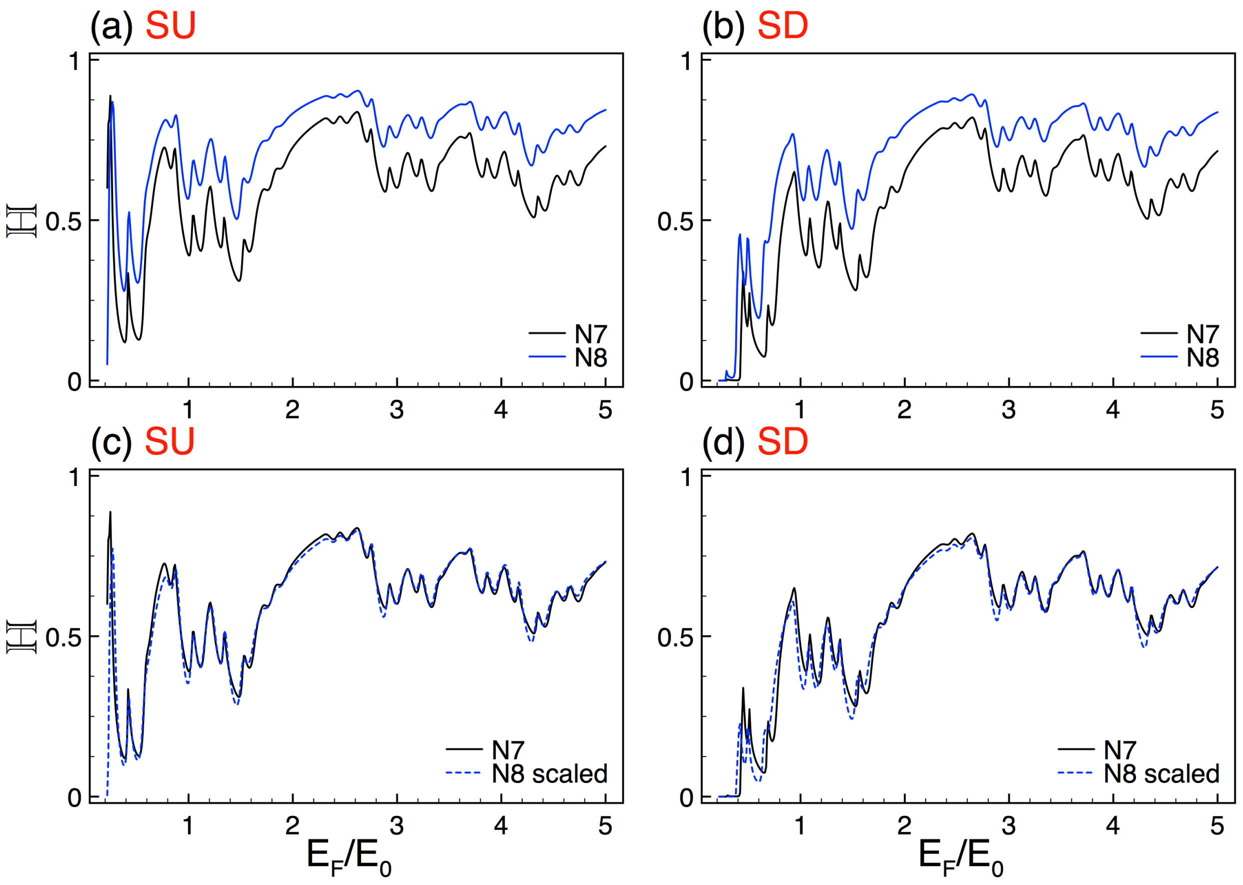

Fig. S7: ℍ scaling between generations for SU and SD. The scaled curve is obtained according to Eq. (S3.2), with $\alpha_{+1} = 1.83$ and $\alpha_{-1} = 1.89$. The panels distribution and structural parameters are the same as in Fig. S1.

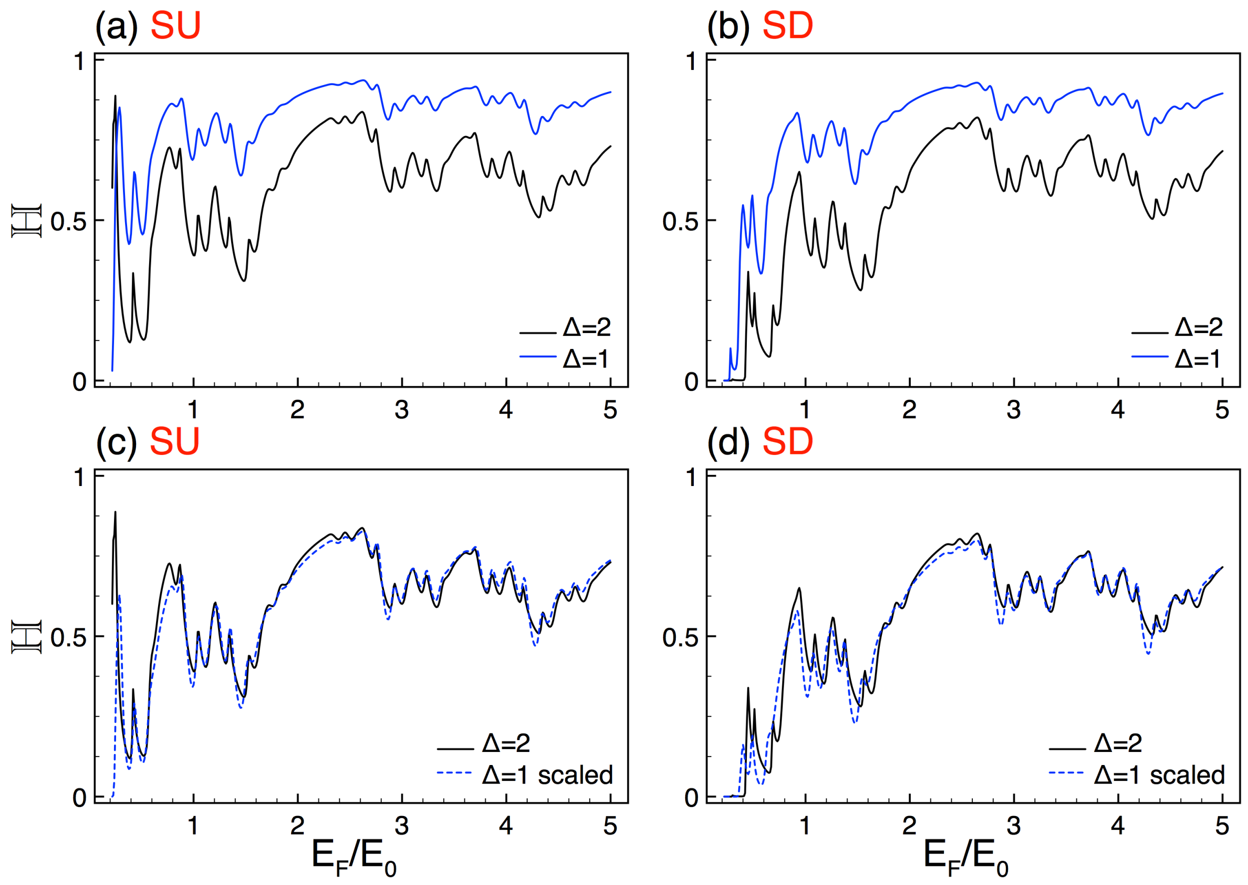

Fig. S8: ℍ scaling between barrier heights for SU and SD. The scaling is calculated using Eq. (S3.3), with $\beta_{+1} = 2.87$ and $\beta_{-1} = 3.02$. The panels distribution and structural parameters are the same as in Fig. S2.

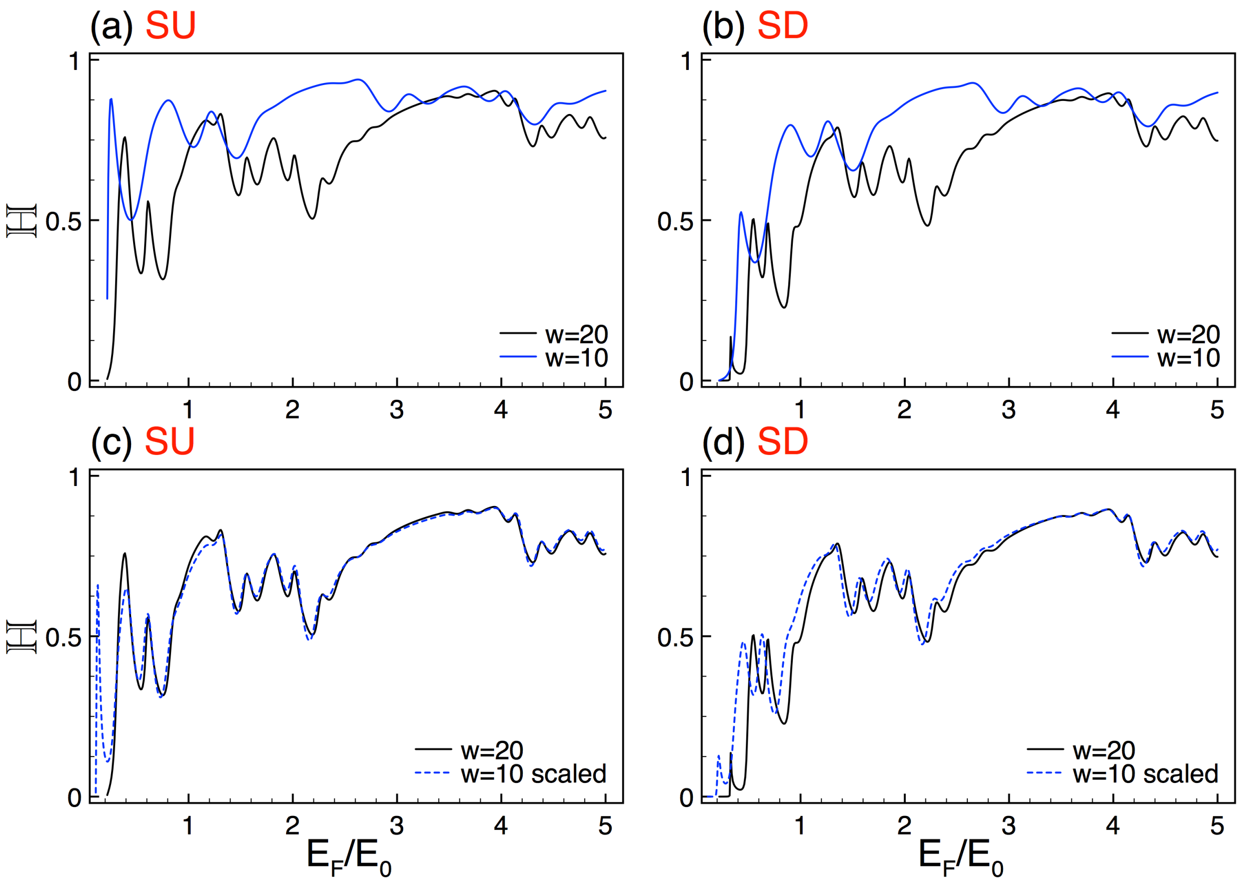

Fig. S9: $\mathbb{H}$ scaling between lengths for SU and SD. The scaled curve is obtained according to Eq. (S3.4), with $\gamma_{+1} = 3.2$ and $\gamma_{-1} = 3.2$. The panels distribution and structural parameters are the same as in Fig. S3.
